# Explainable ensemble deep learning with semantic searching for skin disease classification: a web-based system

**DOI:** 10.3389/fbioe.2026.1885944

**Published:** 2026-10-28

**Authors:** Botheina Hussein, Nora El-Rashidy, Ahmed Taloba, Hela Elmannai, Aishah Alsehaim, Marghny Mohamed

**Affiliations:** 1 Department of Information Systems, Faculty of Computers and Information, Arish University, Arish, Egypt; 2 Department of Information Systems, Faculty of Computers and Information, Assiut University, Asyut, Egypt; 3 Department of Computer Science, University College in Umluj, University of Tabuk, Tabuk, Saudi Arabia; 4 Department of Information Technology, College of Computer and Information Sciences, Princess Nourah bint Abdulrahman University, Riyadh, Saudi Arabia; 5 Department of Computer Science, Faculty of Computers and Information, Assiut University, Asyut, Egypt; 6 Computer Science and Information Technology, Egypt-Japan University of Science and Technology (E-JUST), New Borg El-Arab, Alexandria, Egypt

**Keywords:** convolutional neural network, deep learning, grad-CAM, grad-CAM++, hybrid convolutional neural network–long short-term memory, ISIC 2019 dataset, semantic search, skin disease

## Abstract

Skin diseases are becoming more common around the world. Diagnosing them at an early stage is challenging because of the similarity in shape of different types of lesions and because imaging conditions can vary. This study proposes a unified and lightweight framework for skin lesion classification using the International Skin Imaging Collaboration 2019 dataset by integrating deep learning, stacking ensemble learning, feature embedding-based semantic image retrieval, explainable AI (Grad-CAM and Grad-CAM++), and web-based deployment. The experimental results show that CNN and hybrid CNN–LSTM achieved an accuracy score of 0.9700 and 0.9500, with AUC scores of 0.9987 and 0.9888, respectively. The stacking ensemble achieved high-performance using a XGBoost meta-model, with an accuracy score of 0.9733 and an AUC of 0.9968. While this improvement is modest, the ensemble reduces variance and improves stability and generalization. The main contribution is the integration of classification, case-based semantic retrieval, visual explanation, and practical deployment in a single framework to enable the retrieval of similar images that share the same lesion features as supporting evidence for predictions. Semantic-based retrieval achieved a mAP@5 of 0.981, demonstrating strong feature representations. Since the lightweight architecture has 489,096 parameters, it enables real-time CPU inference and web-based deployment without GPU requirements. Overall, the proposed architecture provides an interpretable, evidence-supported, and practical system for skin lesion classification.

## 1 Introduction

Skin diseases are a common health problem worldwide. Their risks might not always be obvious at first, but they can cause significant physical harm. In critical cases, some skin diseases can lead to skin cancer. Skin disease infections can come from bacteria or viruses, allergic reactions, or other infectious diseases. These diseases often spread quickly through direct contact, making early diagnosis and detection key for effective treatment. Most skin diseases show visible demonstration such as lesions, spots, blisters, or rashes (Al Mamun and Uddin, 2021; Balaji et al., 2020). However, diagnosing the particular disease accurately is difficult because many skin diseases have similar visual features that can make them hard to distinguish. Even skilled dermatologists may make mistaken diagnoses due to differences in lighting, variations in skin color, and differing levels of clinical knowledge (Trager et al., 2022). Skin lesions can be classified into several categories, including actinic keratosis (AK), basal cell carcinoma (BCC), benign keratosis (BKL), dermatofibroma (DF), melanoma (MEL), melanocytic nevus (NV), squamous cell carcinoma (SCC), and vascular lesions (VASC). These lesions look different and vary in severity. Some are temporary, while others are chronic. They can occur with or without pain. Among these types, melanoma is considered the most dangerous and threatens human life. However, about 95% of skin disease patients can be treated if their symptoms are detected early (Vasconcelos and Vasconcelos, 2020). Recently, machine learning (ML) and deep learning (DL) methods have become more common in detecting these diseases. DL models, especially convolutional neural networks (CNNs), have shown great success in detection. CNNs often utilize several pre-trained architectures, including DenseNet121, ResNet50, VGG16, VGG19, AlexNet, and Inception-v3. In several studies, CNNs and transformer frameworks, such as vision and Swin transformers, have been integrated into a single architecture for classifying skin disease images. This architecture extracts local and global spatial features, achieving accuracy scores of 98.30%, 97.89%, and 98.38% on benchmark skin lesion datasets (Jimi et al., 2025; Babu and Madhavi, 2025; Najjar et al., 2025). Howal et al. (2025) combined CNN for feature extraction with SVM for classification. Recently, transformer-based architecture has shown superior performance in skin disease analysis compared with conventional CNN-based approaches; however, such models typically require pre-training on large-scale datasets. Transformers employ multi-head self-attention mechanisms to model long-range dependencies and capture complex relationships among image pixels. For example, Rahman et al. (2025) proposed a ViT-based multi-class skin lesion classification framework which achieved an accuracy of 99.45%.

The main contributions of this study can be summarized as follows.

- Development of a unified lightweight framework for skin lesion classification that integrates CNN, CNN–LSTM, and stacking ensemble model and employs several meta-learners—Random Forest, Extra Trees, AdaBoost, Gradient Boosting, and XGBoost—to achieve competitive performance with a real-time CPU and web-based deployment.
- Integration of semantic search and explainable AI using feature embeddings retrieval, Grad-CAM, and Grad-CAM++ to provide case-based reasoning for similar lesion cases, visual explanations, and evidence-based validation of model predictions.
- Practical and interpretable deployment of the proposed framework, combining accurate classification, case-based reasoning, and explainability within a single web-based system.

The remainder of the study is organized as follows. Section 2 discusses related research, while Section 3 explains the problem statement. Sections 4–6 discuss the dataset, methodology, and data preprocessing steps. Section 7 introduces the proposed approach, while Section 8 discusses the experimental results. Section 9 examines the comparative performance of the proposed models to better understand the results. Finally, the last section concludes with suggestions for future research.

## 2 Related research

A variety of studies have been conducted to improve the detection of skin diseases. These studies focus on DL, ML, and transfer learning. Bipin et al. (2026) proposed a hybrid architecture that integrates ResNet, ConvNeXtV2, and Vision Transformer. This architecture merges the feature extraction of CNN and the global representation learning of transformers on the ISIC 2019 dataset. Initial preprocessing is applied using ResNet-50. Then, local features are extracted using ConvNeXtV2; after that, the ViT-B/16 model captures long-range dependencies across image patches. They applied different data augmentation techniques such as rotation, flipping, zooming, and brightness to improve the model’s generalization. They evaluated their proposed model against transfer learning models such as VGG19 and InceptionV2 and achieved high performance, with an accuracy score of 95.6%.

Naeem et al. (2025) proposed a new DL framework called “SkinDWNet + GB” on the ISIC 2019 dataset. They extracted features by integrating depth-wise dilated convolutions and feature-reuse residual blocks. A gradient boosting classifier was also applied to select the maps of the most informative feature. They applied the SMOTE method to balance the dataset. ANOVA and McNemar’s statistical tests were applied to evaluate the model’s effectiveness. They compared the model’s performance against six transfer learning models, including VGG-19, AlexNet, InceptionV3, DenseNet169, ResNet50, and MobileNetV2. The model achieved the highest accuracy score of 97.04%.

Valarmathi et al. (2025) proposed an ensemble DCNN architecture that integrated three transfer learning models, VGG16, DenseNet121, and Xception, for skin disease detection using two benchmark datasets—ISIC 2020 and HAM10000. They applied different data augmentations such as rotation, zooming, and mirroring to improve the performance and the generalization of the model. DCNN architecture achieved an accuracy score of 94.5% and 83% on the two benchmark datasets, respectively.

Ozdemir and Pacal (2025) proposed a new hybrid DL model using the ISIC 2019 dataset. This model extracted features by integrating ConvNeXtV2 blocks and self-attention mechanisms. The researchers captured local features using ConvNeXtV2 to differentiate between the similar lesion features. Additionally, they applied different data augmentation methods and transfer learning techniques to achieve a robust model to achieve an accuracy score of 93.48%.

Alruwaili and Mohamed (2025) introduced a fusion-based DL architecture combining EfficientNet-B0, EfficientNet-B2, and ResNet50 for skin disease detection on a Kaggle skin diseases dataset. Their architecture applied several data augmentation methods, including rotation, flipping, brightness, and contrast adjustments, to improve the robustness of the model. Their model achieved a top accuracy score of 99.14%, reflecting a significant performance in classification.

Aboulmira et al. (2025) introduced a multimodal fusion framework for skin disease detection using four benchmark datasets: ISIC 2019, ISIC 2020, HAM10000, and PAD-UFES-20. Their framework combined image feature extraction using EfficientNet with tabular data. It used discounted Dempster–Shafer fusion, which included trust-aware evidence weighting and uncertainty modeling. Their results showed the high performance of the proposed framework, with respective accuracy scores of 84.67%, 98.57%, and 94.8% on the three datasets.

Padhy et al. (2025) introduced an innovative hybrid DL model, “Residual Network–Long Short-Term Memory (R-LSTM50)”, evaluated on the ISIC 2020 and HAM10000 datasets. R-LSTM50 integrates ResNet-50 for spatial feature extraction with long short-term memory (LSTM) networks for sequential feature modeling. In addition, advanced data augmentation strategies and weighted loss functions were employed to enhance the model’s performance. R-LSTM50 demonstrated highest accuracies of 95.72% on ISIC 2020 and 94.23% on HAM10000.

Saha et al. (2024) proposed a transformer-based technique for skin cancer classification called “YoTransViT” using the ISIC 2019 dataset. Their system was deployed as a web-based application capable of remotely identifying and diagnosing skin diseases. Their proposed technique integrated vision (ViT) and Swin (SwinViT) transformers with image segmentation based on the YOLOv8 architecture. They evaluated the effectiveness of their model by comparing its performance with three transfer learning models, including MobileNetV2, Inception-ResNetV2, and DenseNet201. The proposed method demonstrated high performance, achieving accuracies of 99.97% and 100%.

Meswal et al. (2024) introduced a weighted ensemble classifier that combined the predictions of seven deep learning models—InceptionV3, VGG16, Xception, ResNet50, MobileNet, a custom CNN, and EfficientNetB—to perform binary classification of melanoma and nevus. Their approach was evaluated on the ISIC 2019 and ISIC 2020 melanoma datasets, achieving an accuracy of 93.36% on ISIC 2019 and 85.54% on ISIC 2020, demonstrating the effectiveness of ensemble learning for melanoma detection across different datasets.

Haghshenas et al. (2024) used vision transformers ViT-B16 and ViT-B32 and transfer learning models Inception-V3 and MobileNet to detect melanoma lesions using the melanoma dataset. Their results showed that the transformers, especially ViT-B16, achieved a high performance, with an accuracy of 97.97%.

Houssein et al. (2024) introduced a new DCNN for skin disease detection. The model was evaluated using ISIC 2019 and HAM10000 datasets, and its performance was evaluated against five pre-trained models: VGG16, VGG19, DenseNet121, DenseNet201, and MobileNetV2. DCNN outperformed and achieved respective accuracy scores of 97.1% and 98.5% on the two datasets.

SM et al. (2023) introduced a skin cancer detection model which also used two benchmark datasets: ISIC 2019 and ISIC 2020. The model was evaluated using several pre-trained models, and it reached an AUC score of 96.81%. A proposed DL-based skin cancer detection architecture, “DSCC_Net” was built based on CNN; it was tested on three benchmark datasets: ISIC 2020, DermIS, and HAM10000. Their proposed model achieved an accuracy of 94.17% in classifying four different types of skin cancer. The effectiveness of DSCC_Net was evaluated through comparative analysis with six transfer learning models—ResNet-152, VGG-19, MobileNet, VGG-16, EfficientNet-B0, and Inception-V3—to achieve accuracies of 89.68%, 92.51%, 91.46%, 89.12%, 89.46%, and 91.82%, respectively.

Keerthana et al. (2023) proposed a binary classification for skin disease images, categorizing them as being either benign or malignant using two novel hybrid CNN-based models that integrate an SVM classifier at the output layer. Features were extracted from the initial and secondary CNN architectures and then fed into the SVM for final classification. The first hybrid model, combining DenseNet-201 and MobileNet, achieved an accuracy of 88.02%, while the second hybrid model, integrating DenseNet-201 and ResNet-50, attained an accuracy of 87.43%.

Hammad et al. (2023) proposed a DL-based model, “Derma Care”, for the binary classification of either eczema or psoriasis using the Eczema and Psoriasis Skin Dataset. Derma Care achieved an accuracy of 96.20%.

Ahammed et al. (2022) proposed a skin disease classification technique based on ML classifiers SVM, KNN, and DT, evaluated on two publicly available benchmark datasets: ISIC 2019 and HAM10000. A customized digital hair removal approach was employed, combining morphological black-hat transformation for hair elimination and Gaussian filtering for image smoothing. Automatic lesion segmentation was performed using the GrabCut algorithm, which effectively identified the disease region. Statistical texture features were extracted using the Gray-Level Co-occurrence Matrix (GLCM). The proposed approach achieved accuracies of 95%, 94%, and 93% on the ISIC 2019 dataset using SVM, KNN, and DT classifiers, respectively, while accuracies of 97%, 95%, and 95% were obtained on the HAM10000 dataset.

Naeem et al. (2022) proposed the DL architecture “SCDNet” which integrated VGG16 with a CNN for multiclass skin cancer classification on the ISIC 2019 dataset, targeting melanoma, basal cell carcinoma (BCC), and benign keratosis (BKL). SCDNet achieved an accuracy of 96.91%, outperforming several transfer learning models: ResNet50 (95.21%), AlexNet (93.14%), VGG19 (94.25%), and Inception-v3 (92.54%).

Kousis et al. (2022) developed and evaluated 11 DL architectures—CNN, AlexNet, VGG16, VGG19, ResNet50, InceptionV3, MobileNetV2, InceptionResNetV2, DenseNet121, DenseNet169, and DenseNet201—for skin disease classification across seven categories—MEL, BCC, AKIEC, NV, BKL, DF, and VASC—and additionally implemented a mobile Android application. Among the models evaluated, DenseNet-169 achieved the highest accuracy of 92.25% compared with the other architectures.

Kaur et al. (2022) proposed an automated melanoma classification system based on a deep convolutional neural network (DCNN) for binary skin tumor classification and evaluated it on the ISIC 2016, ISIC 2017, and ISIC 2020 datasets. The proposed DCNN achieved accuracy of 81.41%, 88.23%, and 90.42% on the ISIC 2016, 2017, and 2020 datasets, respectively.

Xin et al. (2022) introduced a transformer-based architecture for skin cancer classification using a ViT framework on the HAM10000 and a clinical skin cancer dataset. To avoid overfitting, they applied data augmentation methods such as random cropping, random rotation, and color jittering. Their model achieved accuracy scores 94.3% and 94.1% on the two datasets.

The related research is summarized in Table 1.

**TABLE 1:** Related research.

References#	Test	Algorithm	Dataset	Size of dataset	Performance	Limitation
(Bipin et al., 2026)	-	Hybrid (ResNet + ConvNeXtV2 + ViT-B/16)	ISIC 2019	6,200 images are used	Achieved accuracy of 95.6%	Small-scale dataset.
(Naeem et al., 2025)	ANOVA and McNemar’s	DDCs, FRBs, GB	ISIC 2019	25,331 images	Achieved accuracy of 97.04%	Long steps and more time computation
(Valarmathi et al., 2025)	-	Ensemble DCNN	ISIC 2020 and HAM10000	29,126 images, and 10,015 images	Achieved accuracies of 94.5%, and 83% on the two datasets, respectively	More time computation
(Ozdemir and Pacal, 2025)	-	ConvNeXtV2+	ISIC 2019	25,331 images	Achieved accuracy of 93.48%	More time computation
(Alruwaili and Mohamed, 2025)	-	ResNet50, EfficientNet-B0, and EfficientNet-B2	Kaggle Skin Diseases dataset	27,153 images	Achieved accuracy of 99.14%	Long steps and more time computation
(Aboulmira et al., 2025)	-	Dempster–Shafer fusion and EfficientNet	ISIC 2019, ISIC 2020, HAM10000, and PAD-UFES-20	25,331 images, 2,000 images, 10,015 images, and 2,298 clinical images	Achieved accuracy scores of 84.67%, 98.57%, 94.8%, and 90.87% on the four datasets, respectively	Lower performance on the ISIC 2019 dataset.
(Padhy et al., 2025)	-	R-LSTM50(ResNet-50_LSTM)	ISIC 2020 and HAM10000	2,000 images 10,015 images	Achieved accuracies of 95.72% and 94.23% on the two datasets, respectively	Binary classification
(Saha et al., 2024)	-	ViT), Swin transformer (SwinViT	ISIC 2019	25,331 images	Achieved accuracy of 99.97% and 100% precision	Long steps and more time computation
(Meswal et al., 2024)	-	InceptionV3, VGG16, Xception, ResNet50, MobileNet, a custom CNN, and EfficientNetB4	ISIC 2019 and ISIC 2020 melanoma	11,449 images	Achieved accuracy scores of 93.36% and 85.54% in the two datasets	More time computation
(Haghshenas et al., 2024)	-	ViT-B16, ViT-B32, Inception-V3, and MobileNet	Melanoma Dataset	246 images	Vit-B16 achieved the highest accuracy of 97.97%	Small-scale dataset limited to binary classification
(Houssein et al., 2024)	-	DCNN	ISIC-2019 and HAM10000	25,331 images, and 10,015 images	Achieved accuracy scores 97.1% and 98.5%, respectively	Long steps and more time computation
(SM et al., 2023)	-	DCNN	ISIC-2019 and ISIC-2020	25,331 images, 33,126 images	Achieved AUC of 96.81%	Limited set of evaluation metrics
(Tahir et al., 2023)	​	CNN	ISIC 2020, HAM10000, and DermIS	33,126 images, 10,015 images, and 12,345 images	Achieved accuracy of 94.17%	More time computation
(Keerthana et al., 2023)	​	Two hybrid CNN Models, SVM	ISBI 2016 dataset	1,279 images	The two hybrid CNN models achieved accuracy scores of 88.02% and 87.43%	Low performance
(Hammad et al., 2023)	​	CNN	Eczema and Psoriasis Skin Dataset	27,153 images	Achieved accuracy of 96.20%	Binary classification either Eczema or Psoriasis
(Ahammed et al., 2022)	-	SVM, KNN, and DT	ISIC 2019, and HAM10000	25,331 images and 10,015 images	SVM (95%, 97%), KNN (94%, 95%), and DT (93%, 95%) achieved high accuracy on the ISIC 2019 and HAM10000 datasets, respectively	Fails to accurately identify skin lesions using automatic segmentation infrequently
(Naeem et al., 2022)	-	VGG16 + CNN	ISIC 2019	25,331 images	Achieved accuracy of 96.91%	Classification restricted to only four categories
(Kousis et al., 2022)	-	CNN, AlexNet, VGG16, VGG19, ResNet50, InceptionV3, MobileNetV2, InceptionResNetV2, DenseNet121, DenseNet169, DenseNet201	HAM10000	10,015 images	Densenet-169 achieved highest accuracy of 92.25%	Classification restricted to only seven categories
(Kaur et al., 2022)	-	DCNN	ISIC 2016, ISIC 2017, and ISIC 2020	900 images, 2000 images, and 10,070 images	Achieved accuracies of 81.41%, 88.23%, and 90.42% on the	Binary classification
(Xin et al., 2022)	-	VIT	HAM10000, and clinical dataset of skin cancer	10,015 images, 1,016 images	Achieve accuracy of 94.3% and 94.1% in the two datasets	Slow processing performance when handling large images

## 3 Problem statement

Classifying skin diseases is a major challenge in medical diagnosis. Researchers are always trying novel ways to preprocess images and use deep learning (DL) to make diagnosis faster and more accurate. Machine learning (ML) and DL significantly accelerate the diagnosis process and help minimize costs. Several models have been built to address skin diseases using different datasets. However, many of these studies ignore preprocessing steps such as data augmentation and dimensionality reduction, which produce bias and non-trust results. Another challenge is the class imbalance issue. Some skin lesion types may appear in some datasets more than others, which also biases predictions toward those majority classes. In addition, some models require such high resources that they are hard to use in the real world. Other models have complex architectures, low classification accuracy, and need training epochs.

This study addresses these challenges using the following research gaps.

- No prior ISIC 2019 study combines stacking ensemble, semantic search, and Grad-CAM techniques within a single deployable web-based system.
- Class imbalance is addressed using data augmentation rather than simple resampling, which improves data diversity and reduces overfitting while preserving meaningful feature distributions.
- Explainable AI methods such as Grad-CAM and Grad-CAM++ are used to identify lesion regions, which influence the model’s predictions, so doctors can use it to make diagnostic decisions.
- Finally, a semantic search system is needed that is based on feature embeddings which can enable clinicians to identify images that look similar. This can support clinical decision-making.

## 4 Methodology

In this study, we detect skin diseases more effectively using ML and DL. First, the CNN and a hybrid CNN–LSTM are trained, and a stacking ensemble merges the results of these models. We then merge the predictions of the trained models and employ it to train ML classifiers such as Random Forest, Extra Trees, AdaBoost, Gradient Boosting, and XGBoost. Before training, the data is cleaned up, after which it is split into training and testing sets. We also applied the semantic search technique by employing the feature embeddings of the trained models, allowing it to retrieve lesions that look alike by using feature embeddings.

### 4.1 Dataset

The International Skin Imaging Collaboration (ISIC) 2019 benchmark dataset is used in this study (Codella et al., 2019). This dataset has 25,331 dermoscopic images classified into eight classes: actinic keratosis (AK), benign keratosis (BKL), basal cell carcinoma (BCC), dermatofibroma (DF) with 239, melanoma (MEL) with 4,522, melanocytic nevus (NV), vascular lesion (VASC), and squamous cell carcinoma (SCC), as shown in Figure 1, which shows that the dataset is very unbalanced. A few samples from these classes are shown in Figure 2.

**FIGURE 1:**
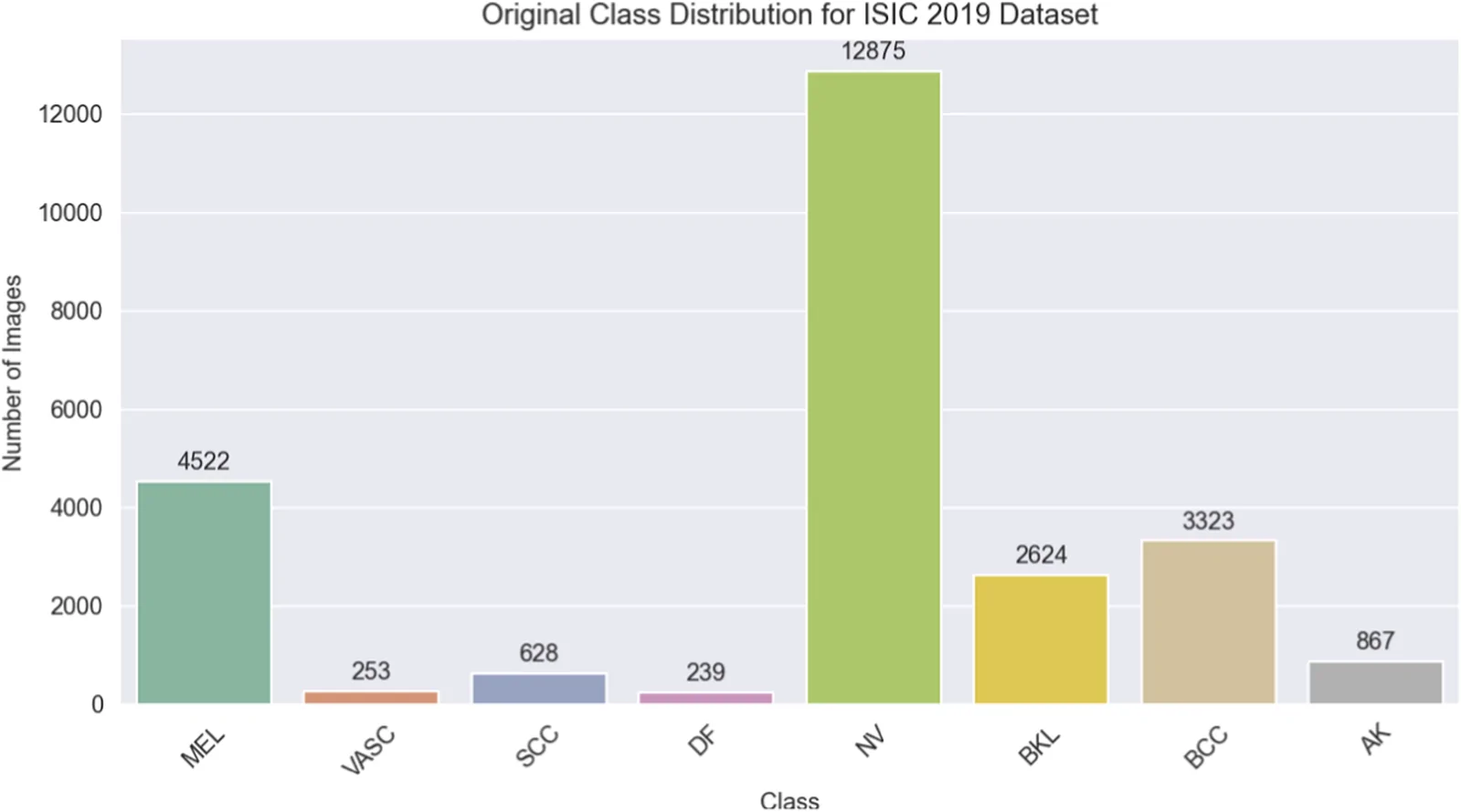
Distribution of classes in the ISIC 2019 dataset.

**FIGURE 2:**
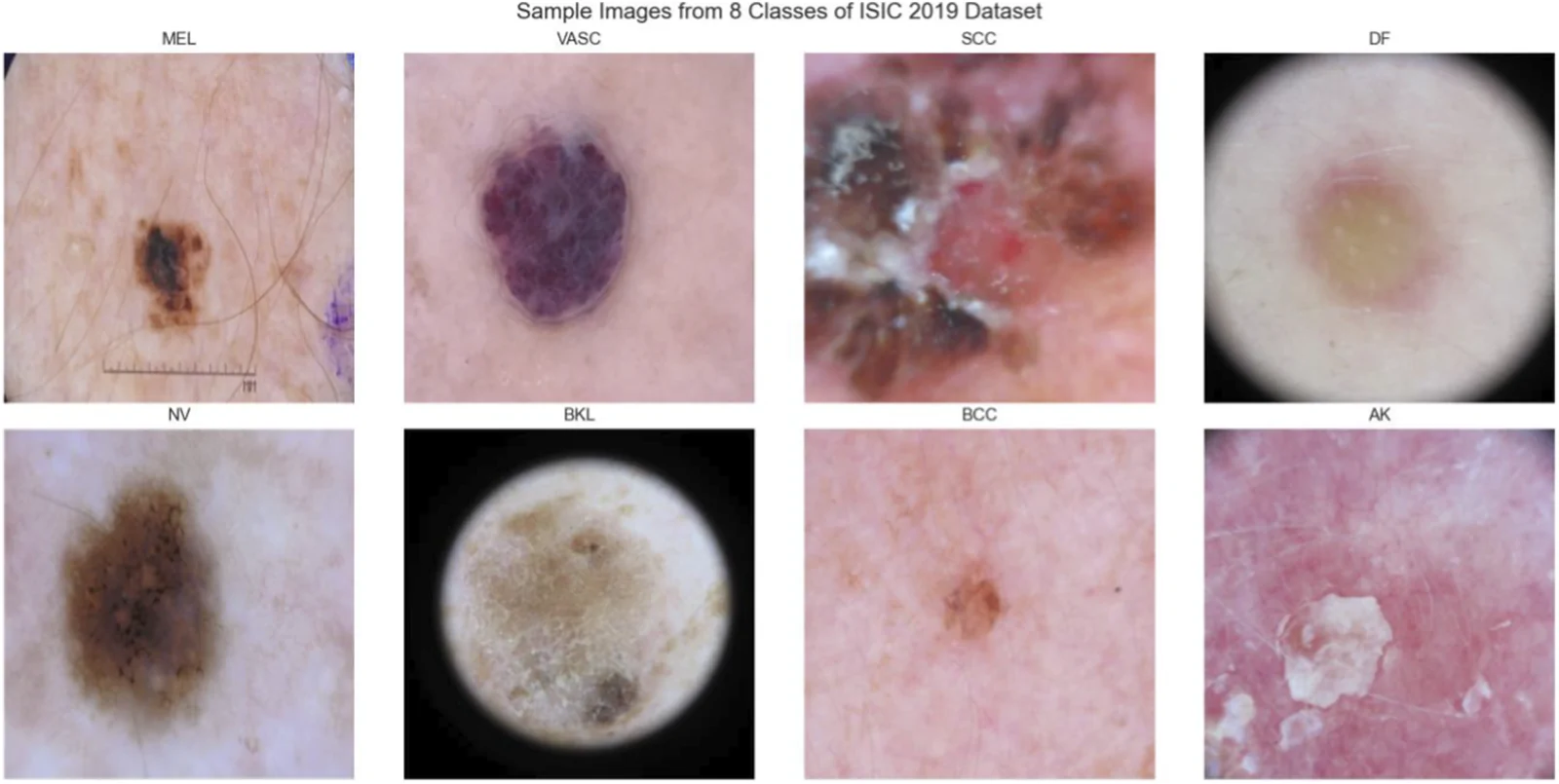
Sample of classes in the ISIC 2019 dataset.

### 4.2 Data preprocessing

In this study, we applied preprocessing and data augmentation methods on the dataset to improve the effectiveness of the trained models. All images were preprocessed by resizing them to 128 × 128 pixels to increase the diversity of the dataset and avoid overfitting. We also applied data augmentation methods such as rotation, flipping, and zooming to data imbalance. Images were rotated by fixed angles of 90°, 120°, and 180°, zoomed in/out by up to 20% zoom range, and randomly flipped horizontally. Contrast was enhanced using Contrast Limited Adaptive Histogram Equalization (CLAHE) (Naeem et al., 2026) to enhance the visibility of lesions in the images. CLAHE was applied with a clip limit of 2.0 and a tile grid size of (8 × 8). The dataset was randomly divided into 80% (82,400 images) for training and 20% (20,600 images) for testing. Data augmentation was then applied only to the training set using these preprocessing techniques (Table 2), thus increasing the dataset from 25,331 to 103,000 images. Due to the class imbalance in the original dataset, these data augmentation methods were mainly applied to the minority classes so that a balanced data distribution of classes is maintained while preserving the original features of the skin images. This balancing method enabled the model to learn features from all classes while reducing the bias to the majority classes. As shown in Table 2, all classes were augmented to 12,875 images, resulting in class balance. Figure 3 shows the distribution of the classes after balancing the dataset through data augmentation.

**TABLE 2:** Class distribution before and after data augmentation.

Class	Original count	Augmented count	Imbalance ratio	Augmentation process
AK	867	12,875	0.067	Flipping (vertical) and rotation (90°, 120°, 180°)
BKL	2,624	12,875	0.204	Flipping (vertical) and CLAHE
BCC	3,323	12,875	0.258	CLAHE
DF	239	12,875	0.019	Flipping (vertical), rotation (90°, 120°, 180°), and zooming (up to 20%)
MEL	4,522	12,875	0.351	Flipping (vertical), rotation (90°, 120°, 180°), and CLAHE
NV	12,875	12,875	1.0	No augmentation applied
VASC	253	12,875	0.020	Flipping (vertical), rotation (90°, 120°, 180°), zooming (up to 20%), and CLAHE
SCC	628	12,875	0.049	Flipping (vertical), zooming (up to 20%), and CLAHE

**FIGURE 3:**
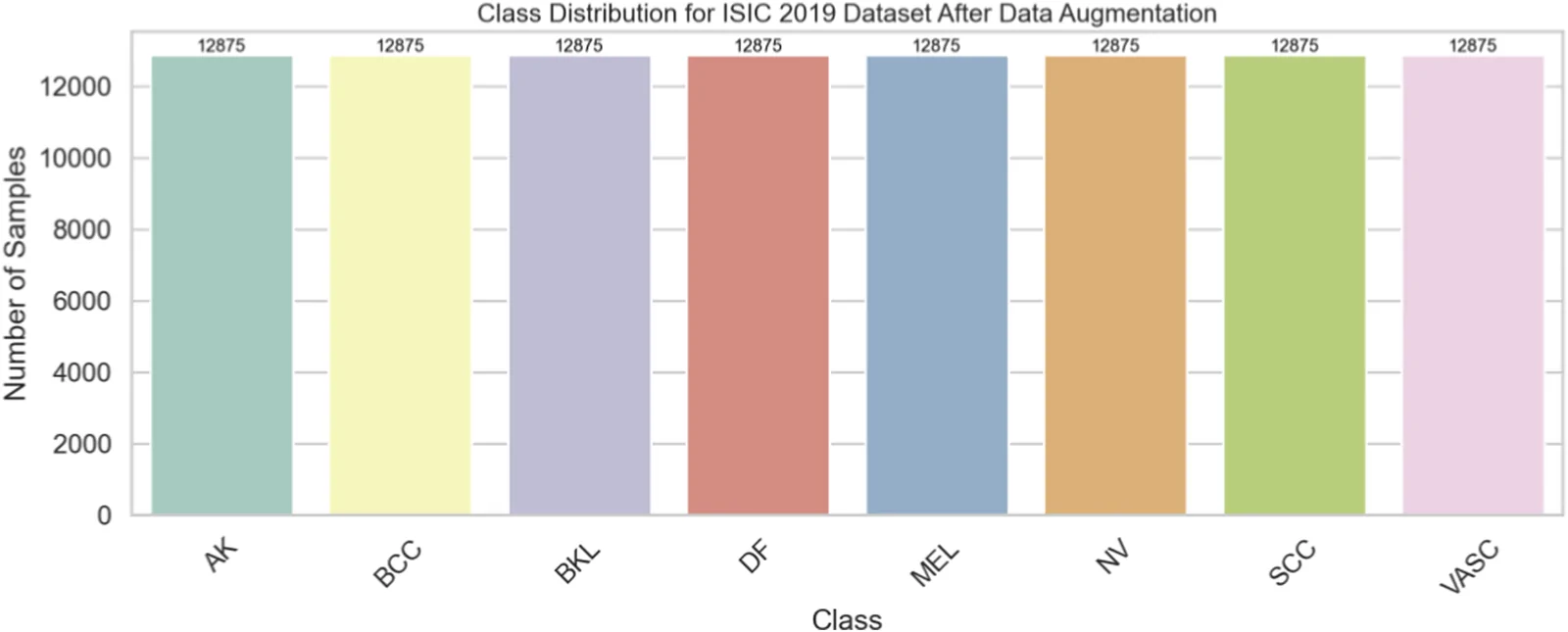
Class distribution after dataset balancing using data augmentation.

## 5 Proposed technique

This study explores how well different DL models can classify skin lesions using the ISIC 2019 dataset. The proposed architecture is designed in several stages (Figure 4). All images were first preprocessed by resizing them to 128 × 128 pixels. These images were then forwarded to the modeling stage, where a CNN model and a hybrid CNN–LSTM model were employed. Their outputs were then combined and fed into ensemble models such as Random Forest, Extra Trees, AdaBoost, Gradient Boosting, and XGBoost. Lastly, different performance metrics were used to evaluate our architecture. To improve the interpretability of the proposed model, Grad-CAM and Grad-CAM++ were employed. In addition, the extracted feature embeddings were utilized for semantic search to retrieve images that share similar features.

**FIGURE 4:**
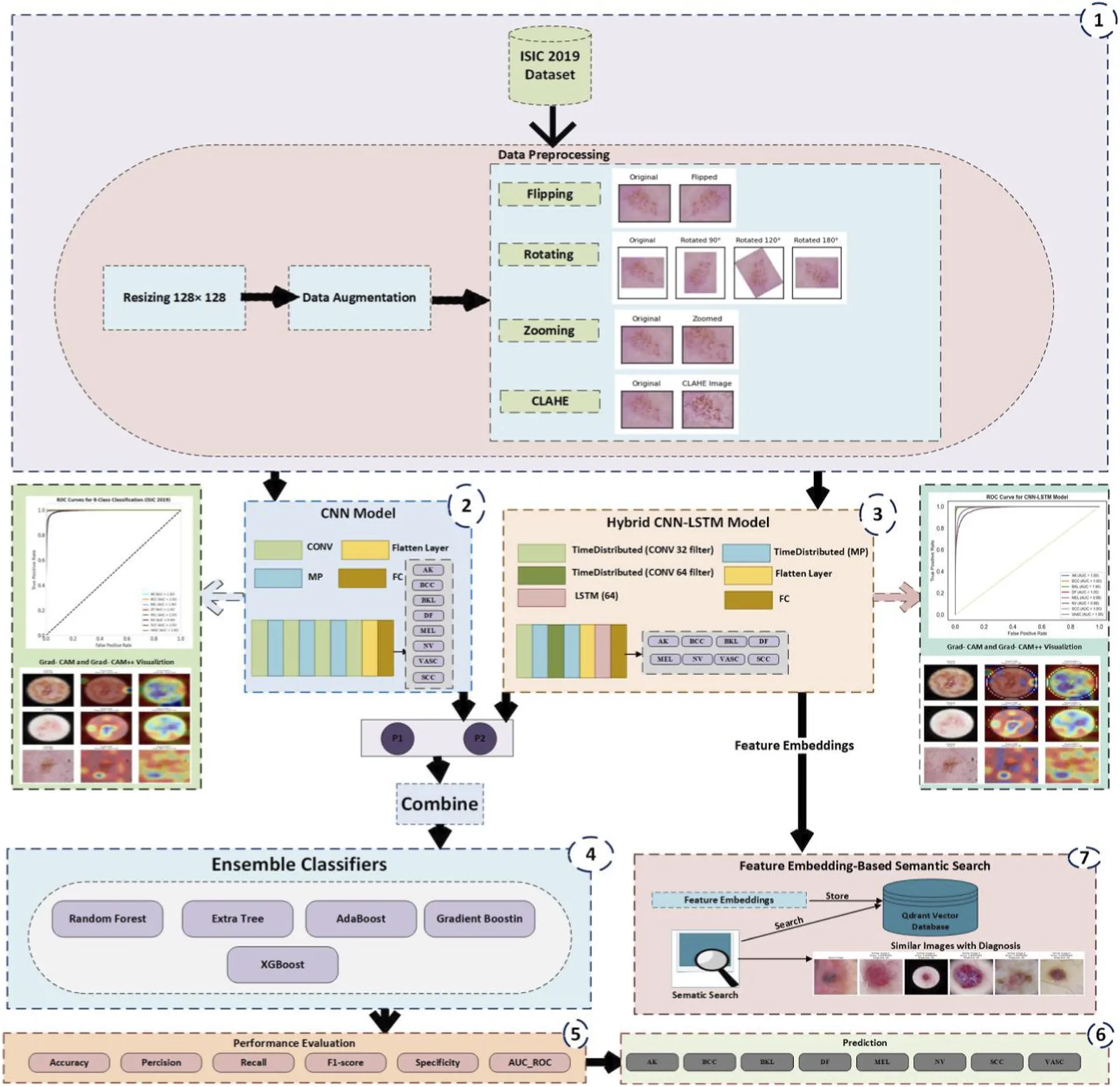
Proposed architecture.

### 5.1 Modeling using CNN

In medical imaging, CNNs have achieved an effective performance in identifying critical visual features in dermoscopic images (He et al., 2019). They are also capable of capturing the differences between types of lesions and handling large, diverse datasets, enabling generalization performance on datasets such as ISIC 2019 (Inthiyaz et al., 2023). The architecture of the proposed model begins with a 128 × 128 × 3 size as an input layer, followed by four conv layers with 32, 64, 128, and 256 filters, respectively. Each conv layer had a kernel size of 3 × 3, ReLU activation, and He-normal initialization, followed by batch normalization and max pooling. After that, to summarize the feature maps, the global average pooling layer was applied. The resulting features were transferred to two fully connected layers of 256 and 128 neurons, each with L2 regularization and dropout to avoid overfitting. Finally, a softmax output layer with eight neurons was used to generate prediction scores for the eight classes. Several quantities of convolutional layers were employed, but four layers provided the best results and minimal model complexity. The Adam optimizer with categorical cross-entropy as a loss function was used in training. The total number of trainable parameters of this proposed model was 489,096, reflecting a relatively lightweight architecture for practical deployment.

### 5.2 Modeling using the hybrid CNN–LSTM model

The proposed hybrid CNN–LSTM model combines the strengths of CNNs and long short-term memory (LSTM) networks to improve dermoscopic image detection. In this hybrid model, three TimeDistributed conv layers with 32, 64, and 128 filters were used as input. Each conv layer had a kernel size of 3 × 3, ReLU activation, and same padding, followed by TimeDistributed max pooling for summarization. After that, the resulting features were flattened using a TimeDistributed layer and then transferred to an LSTM layer with 128 units. This layer captured dependencies through the extracted features. The output was then transferred to fully connected layers with 256 and 128 neurons, along with dropout regularization to improve generalization. Finally, a softmax output layer with eight neurons was used to generate prediction scores for the eight skin lesion classes. This design let the LSTM learn sequences at the feature level, making it useful even though each image was only one timestep. The Adam optimizer with categorical cross-entropy as a loss function was used in training for an accurate classification.

### 5.3 Stacking ensemble model

In this stage, both ML and DL techniques were combined to build an architecture that achieves a good balance between efficiency and prediction efficiency. The outputs from the two previously trained models were merged using a stacking approach and were then used as input features for five ensemble classifiers: Extra Trees, Random Forest, Gradient Boosting, AdaBoost, and XGBoost. To avoid information leakage, out-of-fold predictions were generated from the CNN and CNN–LSTM base models through cross-validation, not in-sample predictions. Training data was split into (k) folds, and the CNN and CNN–LSTM were repeatedly trained on (k-1) folds while the testing was applied on the remaining fold, ensuring that the prediction was based on unseen samples. These out-of-fold predictions were then fed as input features for stacking classifiers. By combining the predictions of the base models, these classifiers enhanced the overall classification performance, leading to an accurate and reliable skin lesion classification.

### 5.4 Grad-CAM and grad-CAM++ for model explainability

Explainable Artificial Intelligence (XAI) plays an important role in interpreting DL models, as these models are often seen as black boxes due to their lack of explainability in decision making. To improve model interpretability, Gradient-weighted Class Activation Mapping (Grad-CAM) and its enhanced version, Grad-CAM++, were applied to visually explain the model’s predictions (Vinogradova et al., 2020). These two methods produce heatmaps that highlight the regions of lesions in image. They work by backpropagating the gradients of the target class score from the final output layer to the feature maps of the last convolutional layer. Grad-CAM identifies the localization maps, while Grad-CAM++ can identify multiple relevant lesions within an image. Their results help visualize skin lesions (Na, 2024).

### 5.5 Feature-embedding-based semantic search

In this stage, the feature embeddings produced by the proposed CNN model are used to retrieve similar images based on similar features. Essentially, the final prediction vectors extracted from the model act as feature representations of the visual characteristics of skin images. These embeddings are stored in Qdrant, a high-performance vector database optimized for efficiently storing and searching high-dimensional data. When a new query image is provided, its feature embedding is generated using the trained CNN model and compared with the embeddings stored in Qdrant. The system then performs a semantic search to retrieve images that share the same visual features. Each retrieved embedding is linked to its corresponding skin image class from the ISIC 2019 dataset. By analyzing these similar features, the system can retrieve images with same visual features, helping the model’s predictions to be more interpretable (Wu, 2020).

## 6 Experimental results

This section provides an overview of the experiments performed on the ISIC 2019 dataset for skin lesion detection. Four different architectures were tested and evaluated: a CNN model, a hybrid CNN–LSTM, a stacking-based ensemble model, and a semantic search framework based on feature embeddings. The results of these methods were also compared with existing studies. Model performance was evaluated using metrics such as accuracy, precision, recall, and F1- and AUC scores to offer an effective approach for classifying the various skin lesion classes.

### 6.1 Experimental setup

A workstation running Windows Server 2022 Standard Evaluation (64-bit) with two CPUs and 256 GB of RAM was used to conduct the experiments. TensorFlow, scikit-learn, and Seaborn were used to build, train, and test the models, which were created and run in Python using Spyder 3.13.

### 6.2 Results

To select the model with the best predictive capability for skin lesion detection, the performance of four experiments was evaluated mainly based on accuracy. The following subsections show the results and the comparative evaluations of the proposed models on the ISIC 2019 dataset.

#### 6.2.1 Results of CNN model

This section presents the results of the proposed CNN model as summarized in Table 3. It achieved the highest accuracy score of 0.97. It also achieved the highest F1-score, with a high score of 1.00 for AK, DF, SCC, and VASC, 0.97 for BCC and BKL, 0.92 for MEL, and 0.87 for NV. This model demonstrated excellent performance; it also achieved an AUC of 1.00 for most classes and 0.99 for MEL and NV. Figures 5–7 present the confusion matrix, training and validation curves, and AUC curves. These confirm the model’s strong performance and near-perfect AUC scores, highlighting the robustness of the proposed architecture.

**TABLE 3:** Evaluation metrics of proposed CNN model.

Algorithm	Skin Disease_Stage	Accuracy	Precision	Recall	f1-score	Support	Specificity	AUC_ROC
CNN	AK	0.97	1.00	1.00	1.00	2,582	1.00	1.0
	BCC		0.95	0.99	0.97	2,665	0.99	1.0
	BKL		0.95	0.99	0.97	2,582	0.99	1.0
	DF		1.00	1.00	1.00	2,517	1.00	1.0
	MEL		0.91	0.93	0.92	2,612	0.98	0.99
	NV		0.94	0.81	0.87	2,557	0.99	0.99
	SCC		1.00	1.00	1.00	2,550	1.00	1.0
	VASC		1.00	1.00	1.00	2,535	1.00	1.0

**FIGURE 5:**
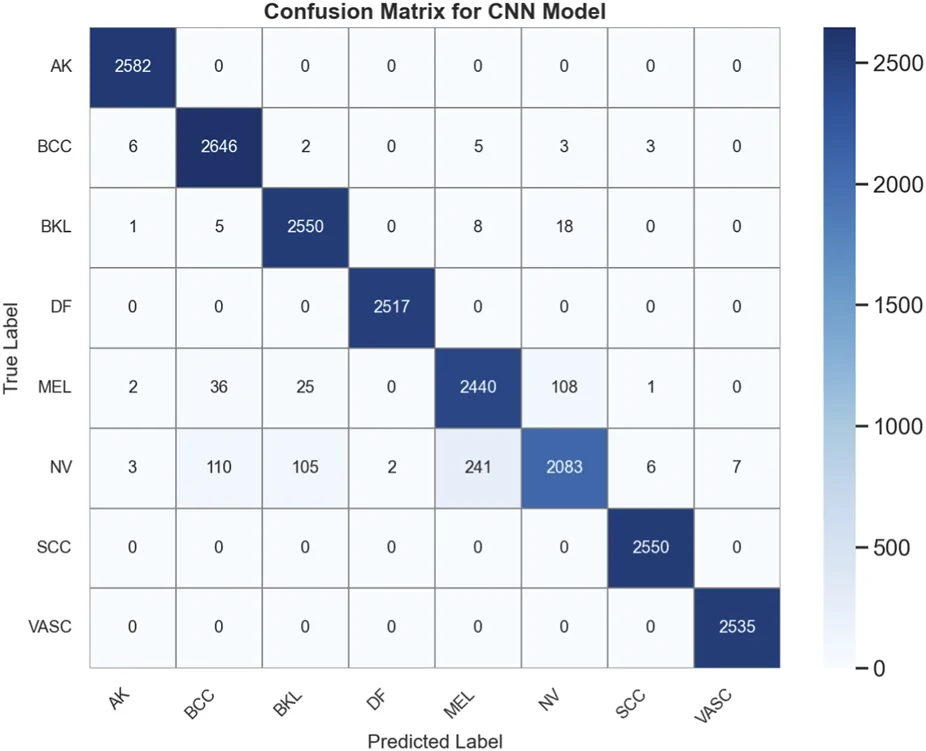
Confusion matrix of the proposed CNN model.

**FIGURE 6:**
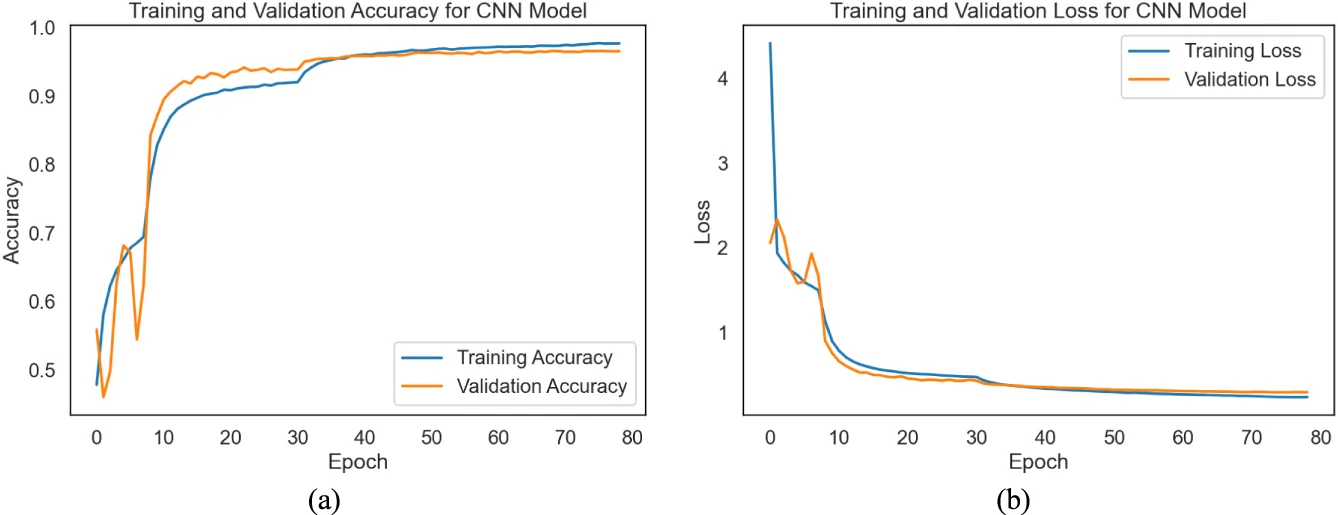
Training and validation accuracy and loss on the ISIC 2019 dataset using the CNN model: **(a)** training and validation accuracy; **(b)** training and validation loss.

**FIGURE 7:**
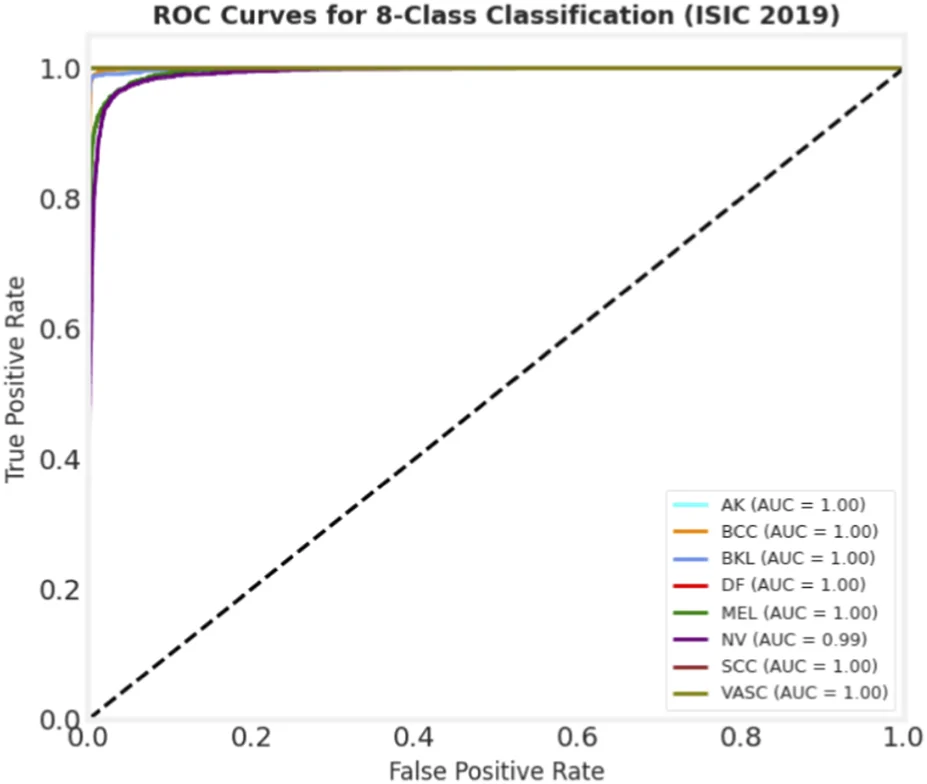
ROC curves for the ISIC 2019 dataset using CNN model.

#### 6.2.2 Results of hybrid CNN-LSTM model

This section presents the evaluation performance of the hybrid CNN–LSTM model as summarized in Table 4. This model’s highest accuracy score was 0.95. The F1-scores reached 1.0 with AK, DF, and VASC, 0.99 for SCC, 0.97 for BCC, 0.95 for BKL, 0.87 for MEL, and 0.80 for NV. It also showed strong performance with most classes, achieving an AUC score of 1.00, while MEL and NV have scores of 0.99 and 0.98. The confusion matrix (Figure 8) provides detailed classification results across all classes. Figure 9 displays the training and validation accuracy and loss curves, which slowly increase during the early epochs and then converge closely, indicating strong learning with minimum overfitting. Figure 10 presents the AUC curves for all eight classes, where most AUCs reached 1.0, indicating the robustness and generalization of the hybrid CNN–LSTM model.

**TABLE 4:** Classification metrics of proposed hybrid CNN–LSTM Model.

Algorithm	Skin Disease_Stage	Accuracy	Precision	Recall	f1-score	Support	Specificity	AUC_ROC
CNN	AK	0.95	0.99	1.00	1.00	2,582	1.00	1.0
	BCC		0.95	0.98	0.97	2,665	0.99	1.0
	BKL		0.91	0.99	0.95	2,582	0.99	1.0
	DF		1.00	1.00	1.00	2,517	1.00	1.0
	MEL		0.86	0.88	0.87	2,612	0.98	0.99
	NV		0.88	0.73	0.80	2,557	0.99	0.98
	SCC		0.99	1.00	0.99	2,550	1.00	1.0
	VASC		1.00	1.00	1.00	2,535	1.00	1.0

**FIGURE 8:**
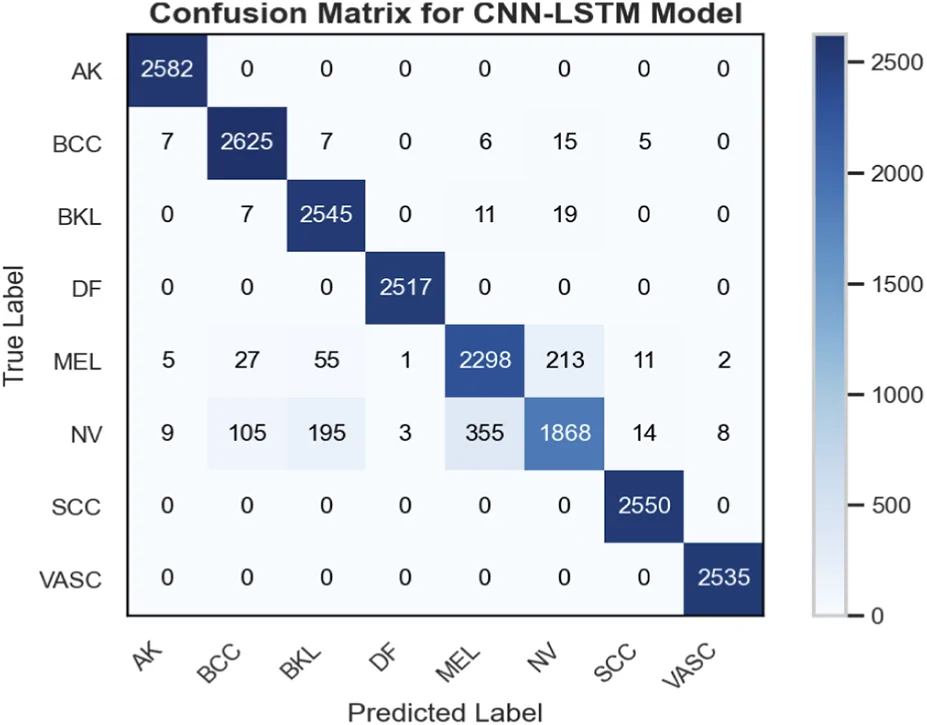
Confusion matrix of the proposed hybrid CNN-LSTM model.

**FIGURE 9:**
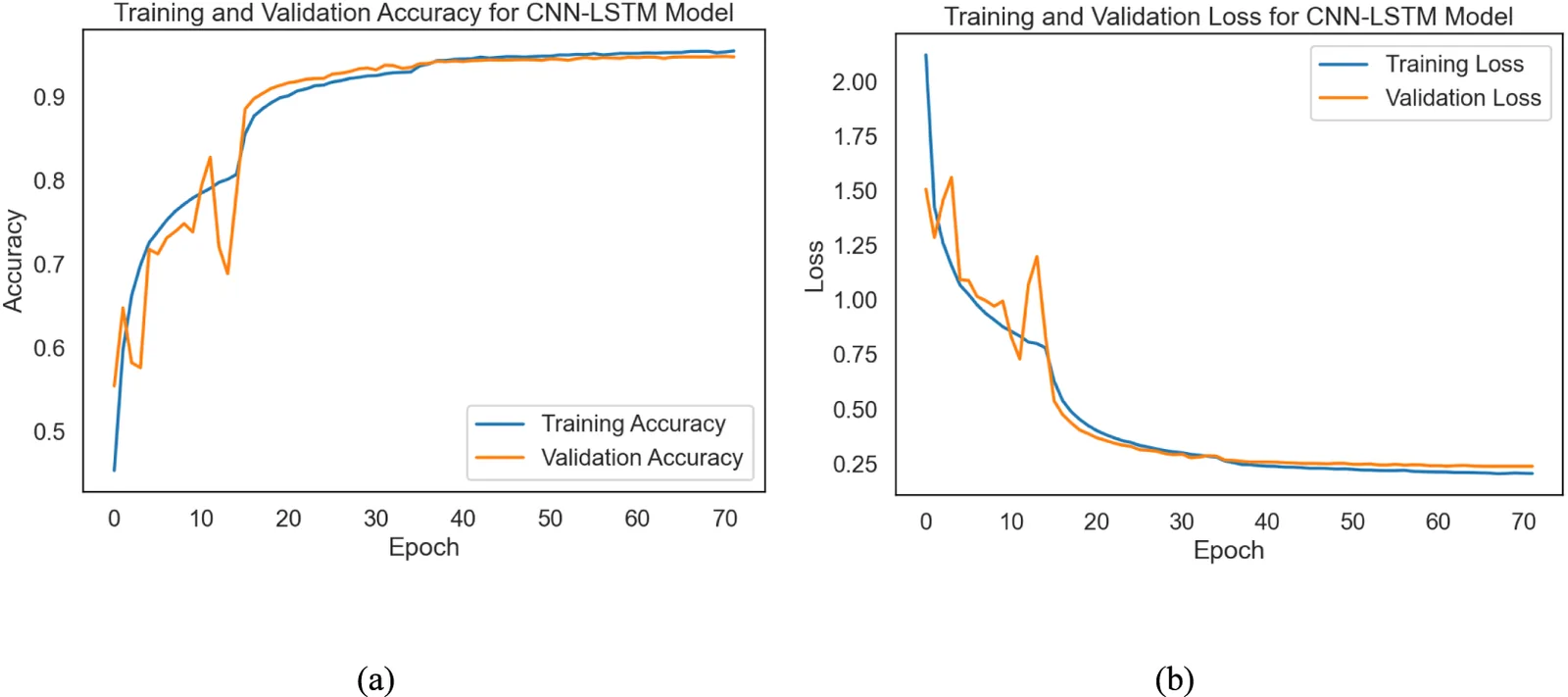
Training and validation accuracy and loss on the ISIC 2019 dataset using the hybrid CNN-LSTM model: **(a)** training and validation accuracy; **(b)** training and validation loss.

**FIGURE 10:**
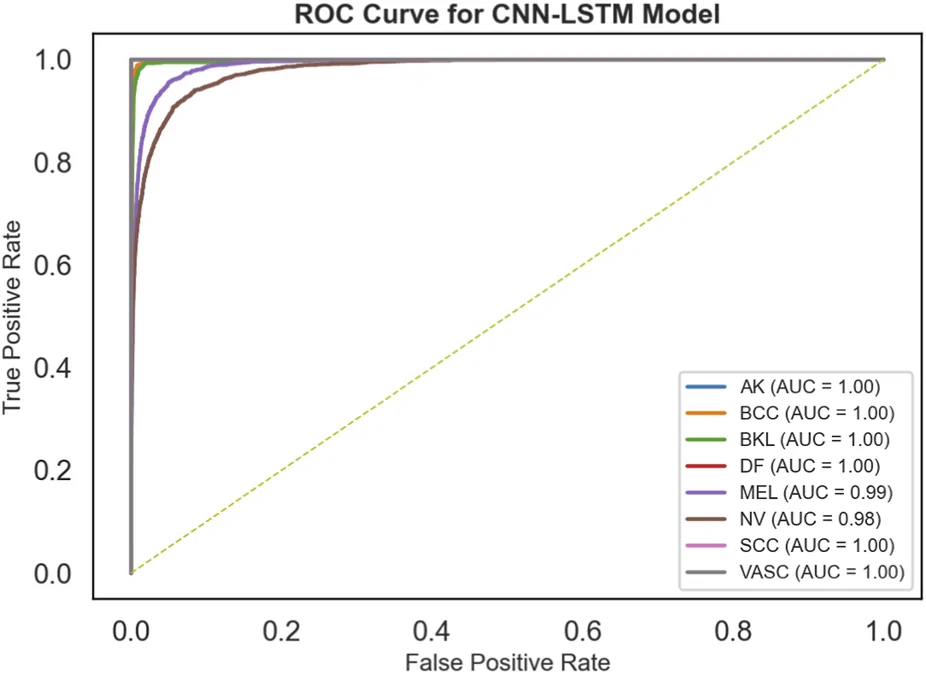
ROC curve for ISIC 2019 dataset using the hybrid CNN-LSTM model.

#### 6.2.3 Results of stacking ensemble model

We utilized the predictions of the previous proposed models as inputs to train six ensemble classifiers, including Random Forest, Extra Trees, AdaBoost, Gradient Boosting, and XGBoost, to improve the overall performance. Their results are presented in Table 5. Among them, the XGBoost model achieved an accuracy score 0.9733 and an F1-score of 1.00 for AK, DF, SCC, and VASC, 0.98 for BCC and BKL, 0.93 for MEL, and 0.90 for NV, and an average AUC of 0.9968. Extra Trees classifier achieved a very similar accuracy score of 0.9730 and achieved an average AUC score of 1.0. AdaBoost followed them, achieving an accuracy score of 0.9728 and an average AUC score of 0.9972. Gradient Boosting acquired an accuracy score of 0.9723 with an average AUC score of 0.9965, while Random Forest also performed strongly, achieving an accuracy score of 0.9716 and an average AUC score of 0.9962. The AUC curves are shown in Figure 11, indicating that most classifiers achieved strong learning performance for several classes, particularly with classes AK, DF, SCC, and VASC. However, we observed more challenges in classifying the MEL and NV classes. Figure 12 provides a comparison among the ensemble models, indicating that XGBoost and Extra Trees offer the best performance, especially for accuracy and AUC. Generally, their results showed that ensemble models improved classification performance.

**TABLE 5:** Evaluation metrics of ensemble classifiers using stacking ensemble model.

Algorithm	Skin Disease_Stage	Accuracy	Precision	Recall	f1-score	Specificity	Average AUC_ROC
Random Forest	AK	0.9716	1.0	1.00	1.00	1.0	0.9962
	BCC		0.98	0.98	0.98	0.99	
	BKL		0.97	0.99	0.98	0.99	
	DF		1.00	1.00	1.00	1.0	
	MEL		0.92	0.93	0.93	0.99	
	NV		0.93	0.87	0.00	0.99	
	SCC		0.99	1.00	1.0	1.0	
	VASC		1.0	1.0	1.0	1.0	
Extra Tree classifier	AK	0.9730	1.0	1.0	1.0	1.0	1.0
	BCC		0.98	0.98	0.98	0.99	
	BKL		0.97	0.99	0.98	0.99	
	DF		1.0	1.0	1.0	1.0	
	MEL		0.92	0.93	0.93	0.99	
	NV		0.92	0.88	0.90	0.99	
	SCC		1.0	1.0	1.0	1.0	
	VASC		1.0	1.0	1.0	1.0	
Gradient Boosting	AK	0.9723	1.0	1.0	1.0	1.0	0.9965
	BCC		0.98	0.99	0.98	0.99	
	BKL		0.97	0.99	0.98	0.99	
	DF		1.0	1.0	1.0	1.0	
	MEL		0.91	0.93	0.92	0.99	
	NV		0.92	0.87	0.90	0.99	
	SCC		1.0	1.0	1.0	1.0	
	VASC		1.0	1.0	1.0	1.0	
AdaBoost	AK	0.9728	1.0	1.0	1.0	1.0	0.9972
	BCC		0.98	0.98	0.98	0.99	
	BKL		0.97	0.99	0.98	0.99	
	DF		1.0	1.0	1.0	1.0	
	MEL		0.92	0.93	0.92	0.99	
	NV		0.92	0.88	0.90	0.99	
	SCC		1.0	1.0	1.0	1.0	
	VASC		1.0	1.0	1.0	1.0	
XGBoost	AK	0.9733	1.0	1.0	1.0	1.0	0.9968
	BCC		0.98	0.99	0.98	0.99	
	BKL		0.97	0.99	0.98	0.99	
	DF		1.0	1.0	1.0	1.0	
	MEL		0.92	0.93	0.93	0.99	
	NV		0.93	0.88	0.90	0.99	
	SCC		1.0	1.0	1.0	1.0	
	VASC		1.0	1.0	1.0	1.0	

**FIGURE 11:**
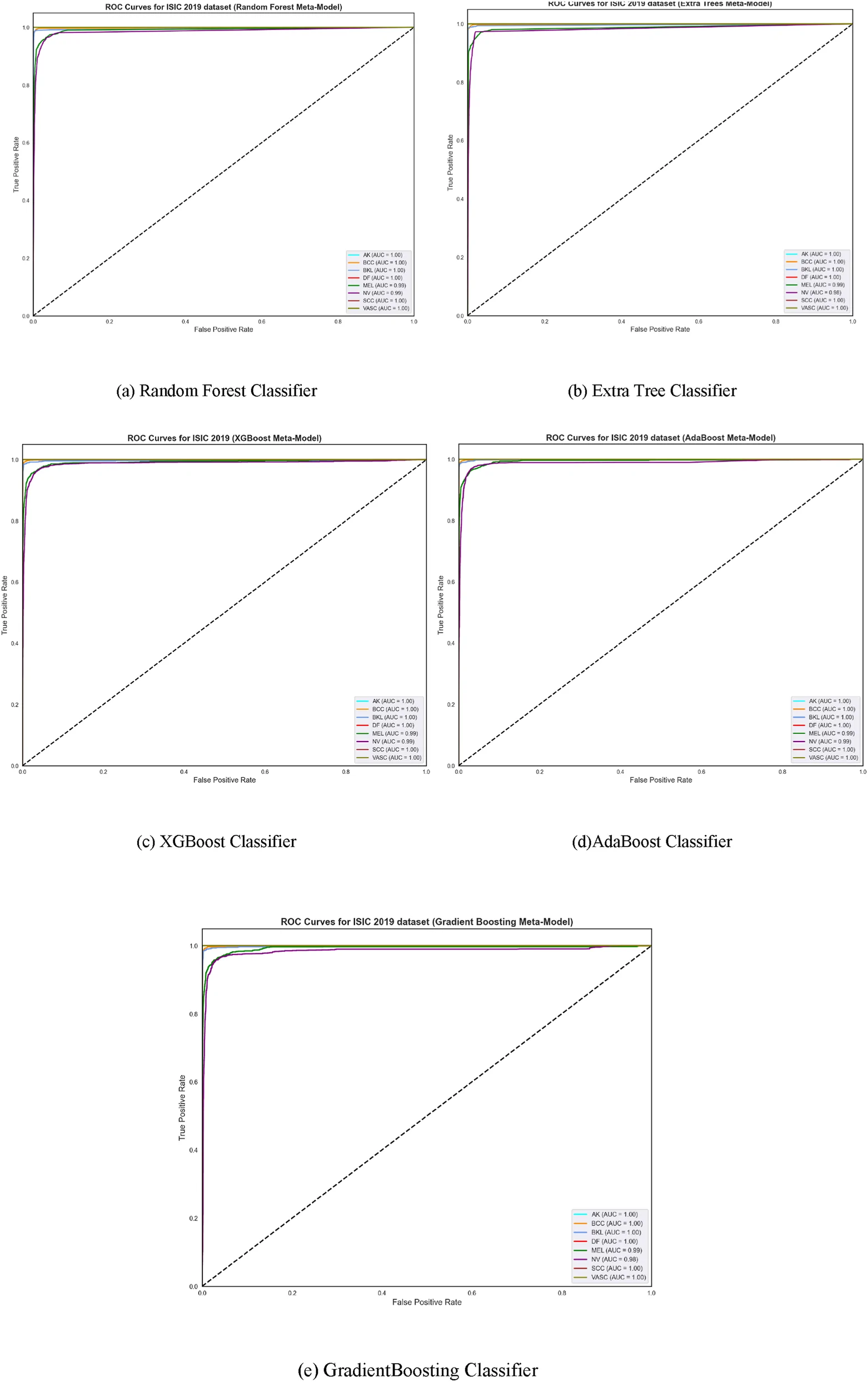
AUC curves of ensemble classifiers across the ISIC 2019 dataset. **(a)** Random Forest classifier. **(b)** Extra Tree classifier. **(c)** XGBoost classifier. **(d)** AdaBoost classifier. **(e)** GradientBoosting classifier.

**FIGURE 12:**
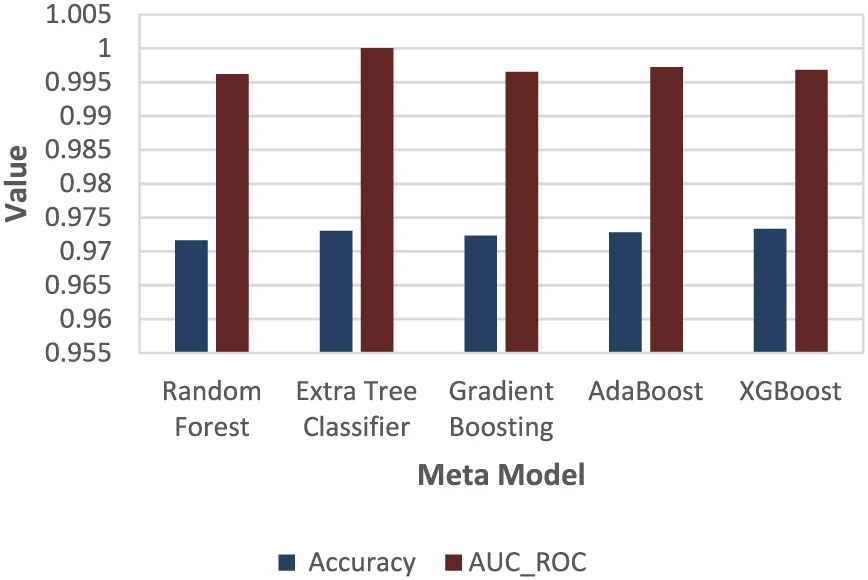
Comparison of ensemble stacking models.

#### 6.2.4 Grad-CAM- and Grad-CAM++-based explainability analysis

Figure 13 shows the Grad-CAM and Grad-CAM++ visualizations for some sample images from the ISIC 2019 dataset. Each row shows the original image beside the heatmaps of Grad-CAM and Grad-CAM++ and the predicted class and confidence score. The heatmaps highlight the influence of the model from low (blue) to high (yellow) and were generated using the last convolutional layer of the CNN model. Overall, these methods highlighted the lesion areas affected by the trained model, supporting the interpretability of the model. For example, in MEL class, both Grad-CAM and Grad-CAM++ clearly highlight the lesion areas, while for other classes such as DF and VASC, the highlighted areas tend to be less obvious. The high confidence score indicates that the model makes predictions based on the relevant lesion features. However, a limitation of this analysis is that we cannot quantitatively evaluate the resultant heatmaps, such as by measuring the intersection over union (IoU) against ground-truth lesion masks. This is because all images in the ISIC 2019 dataset do not include segmentation masks. Therefore, we rely on visual inspection of the results to assess localization quality.

**FIGURE 13:**
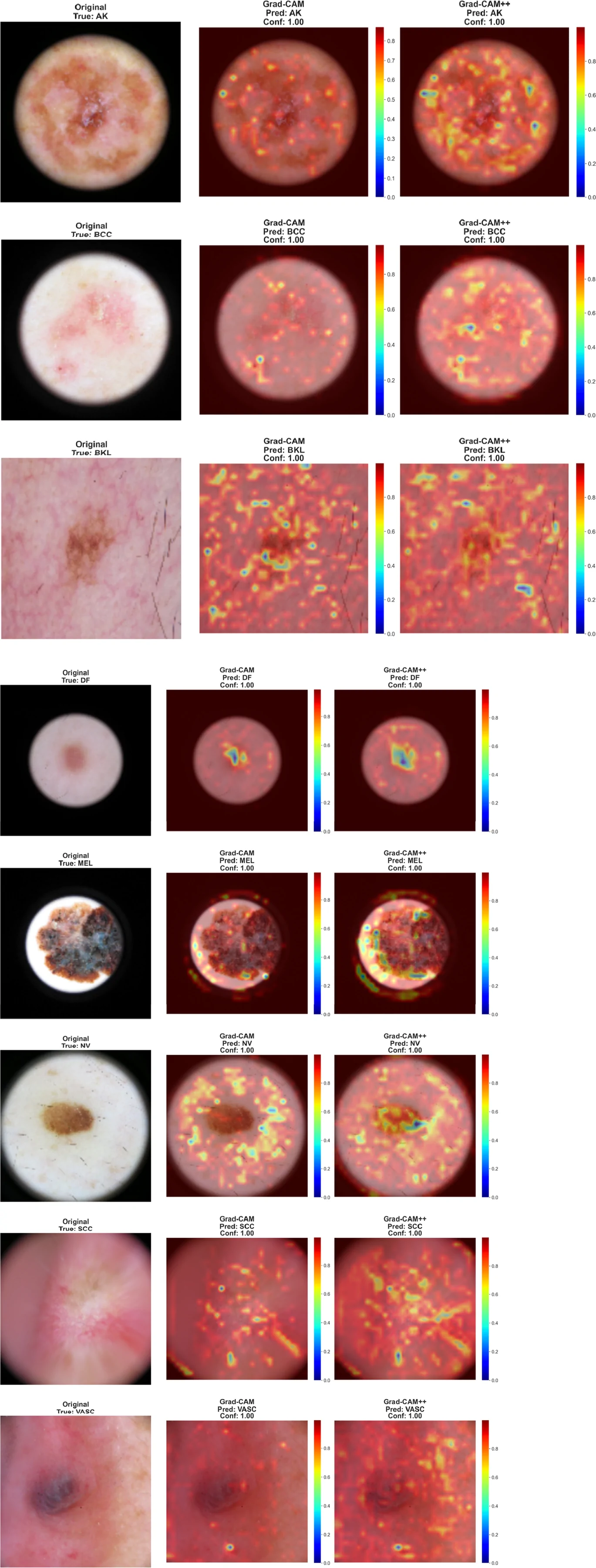
Visualizations of Grad-CAM and Grad-CAM++ for the ISIC 2019 dataset.

#### 6.2.5 Results of feature-embedding-based semantic search model

In this approach, a CNN model is used to extract feature embeddings from its predictions, where it captures the visual features. The cosine similarity approach is applied to determine the similarity between two images by measuring vectors that are close to each other. The images that have vectors similar to the query vector are the best retrieved matches. When we query for an image, the same trained model is employed to convert the query image to a feature embedding. This feature embedding is then compared with those stored in the Qdrant database. Qdrant uses the Approximate Nearest Neighbor (ANN) algorithm to quickly retrieve similar matches. According to this, the system can retrieve some images that share the same features with the query image. For instance, if the query image represents the BCC class, the system selects the top five most similar images which belong to BCC. This shows that the model can recognize images with similar visual features. The retrieved results demonstrate that feature-embedding-based semantic searching is effective, accurate, and reliable for retrieving relevant skin lesion images (Figure 14).

**FIGURE 14:**
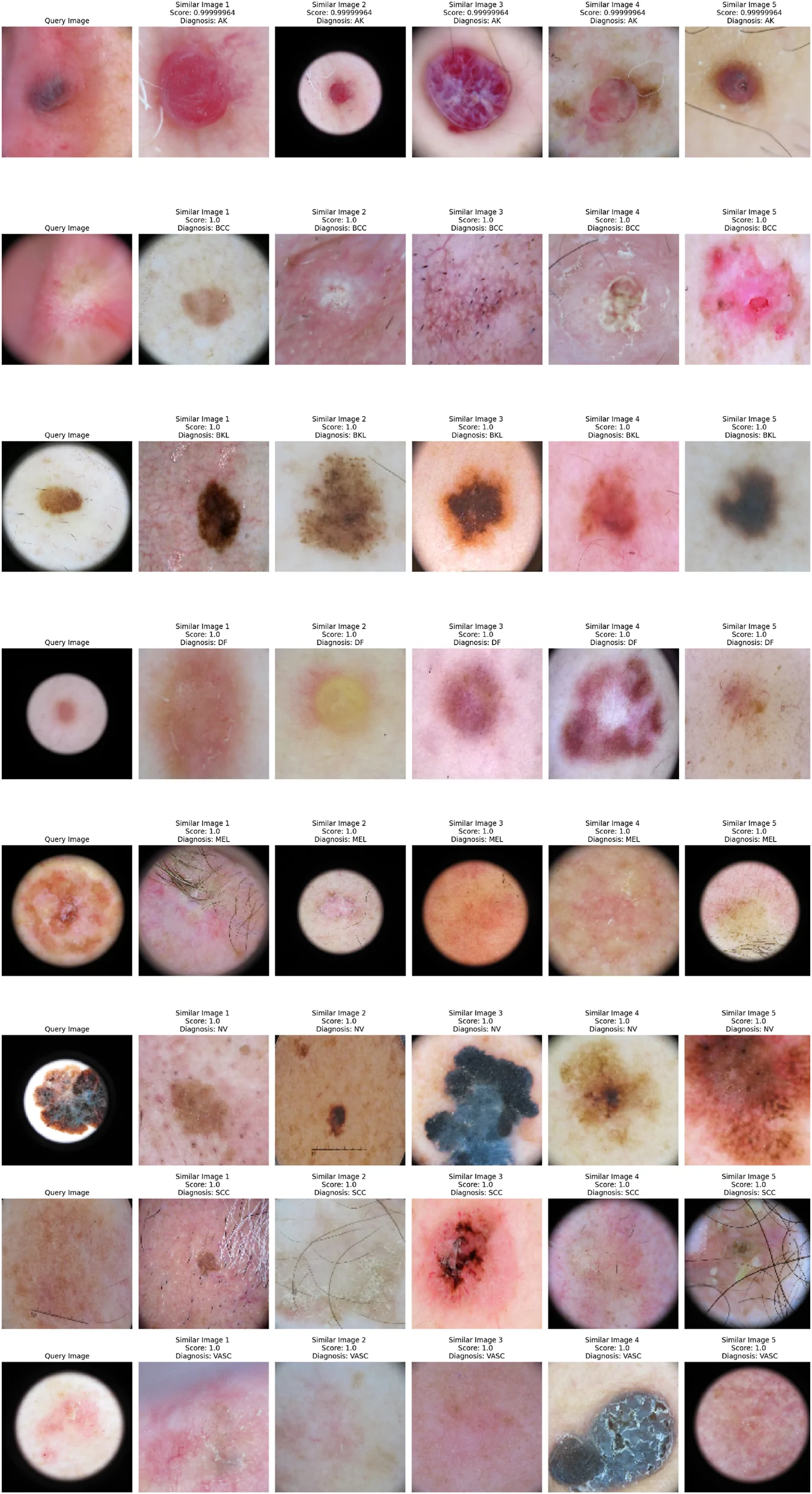
Sample results of feature-embeddings-based semantic search for all ISIC 2019 classes.

#### 6.2.6 Web-based application

We introduced a web-based classification system for skin diseases by integrating our proposed DL models with web-based frameworks. The backend of the system is implemented using the Flask framework. It provides a user-friendly interface with responsive interaction. The system analyzes the uploaded image and generates its classification class, such as actinic keratosis, benign keratosis, melanoma, and the other classes included in the ISIC 2019 dataset. Our system aims to enable dermatologists’ decision-making in diagnosing skin disease, to detect the disease at early stage, and help lower treatment costs by providing reliable diagnosing and fast decisions to both medical professionals and users. Each image is analyzed in less than 0.5 s on the CPU, demonstrating a rapid response suitable for clinical use. To guarantee security and privacy, uploaded images are analyzed temporarily and not stored. This allows patient data to be saved during analysis. However, the system has not yet gone through any kind of external validation, prospective validation, or clinical validation. For this reason, we need more clinical validation of the system before deployment in actual clinical practice to evaluate its usefulness in helping patients self-diagnose, lowering treatment costs, and making clinical decisions. The system is publicly available at: https://github.com/DrBothaina/Skin_Disease_Detection. Figure 15 illustrates the homepage of our system, where users can click the “Classify Skin Lesion” button to move to the classification page (Figure 16). Here, users can upload their image for analysis. Once the image is uploaded, the system displays the predicted disease along with its accuracy percentage (Figure 17). The “skin diseases” page presents more information about the different skin diseases as shown in Figure 17, while the “Contact Us” page allows users to submit their feedback.

**FIGURE 15:**
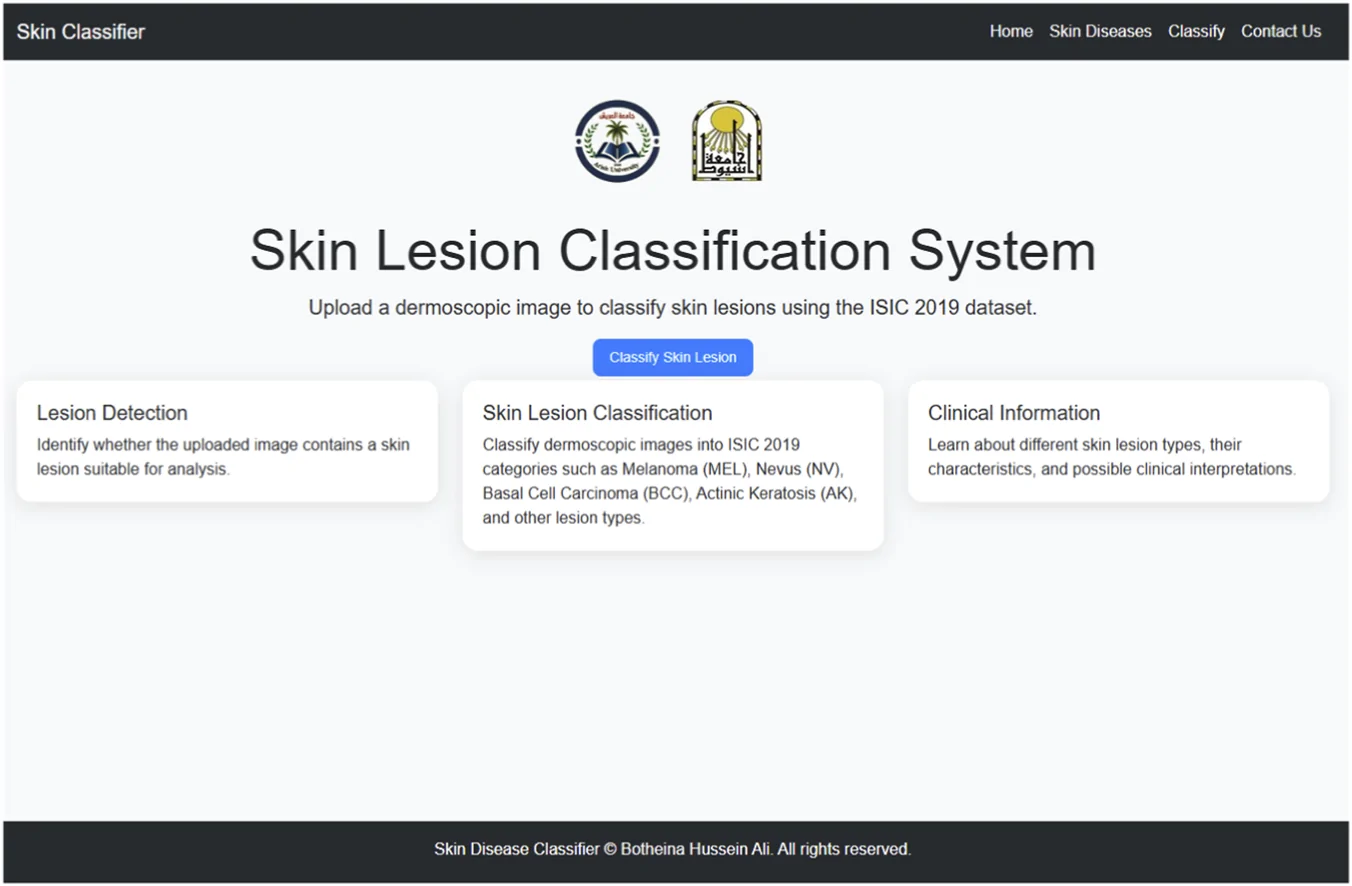
Proposed web-based system home page.

**FIGURE 16:**
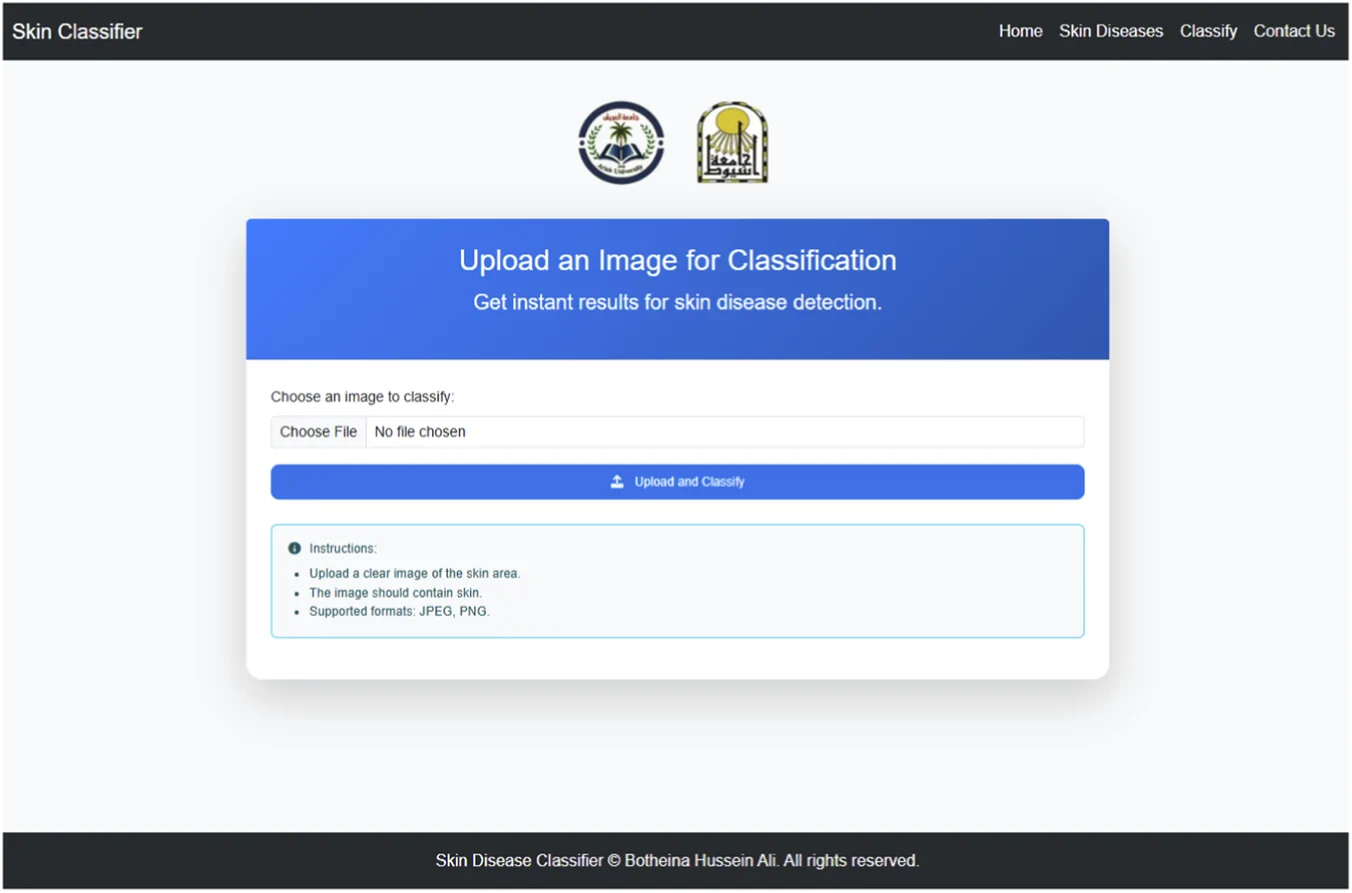
“Classify” page.

**FIGURE 17:**
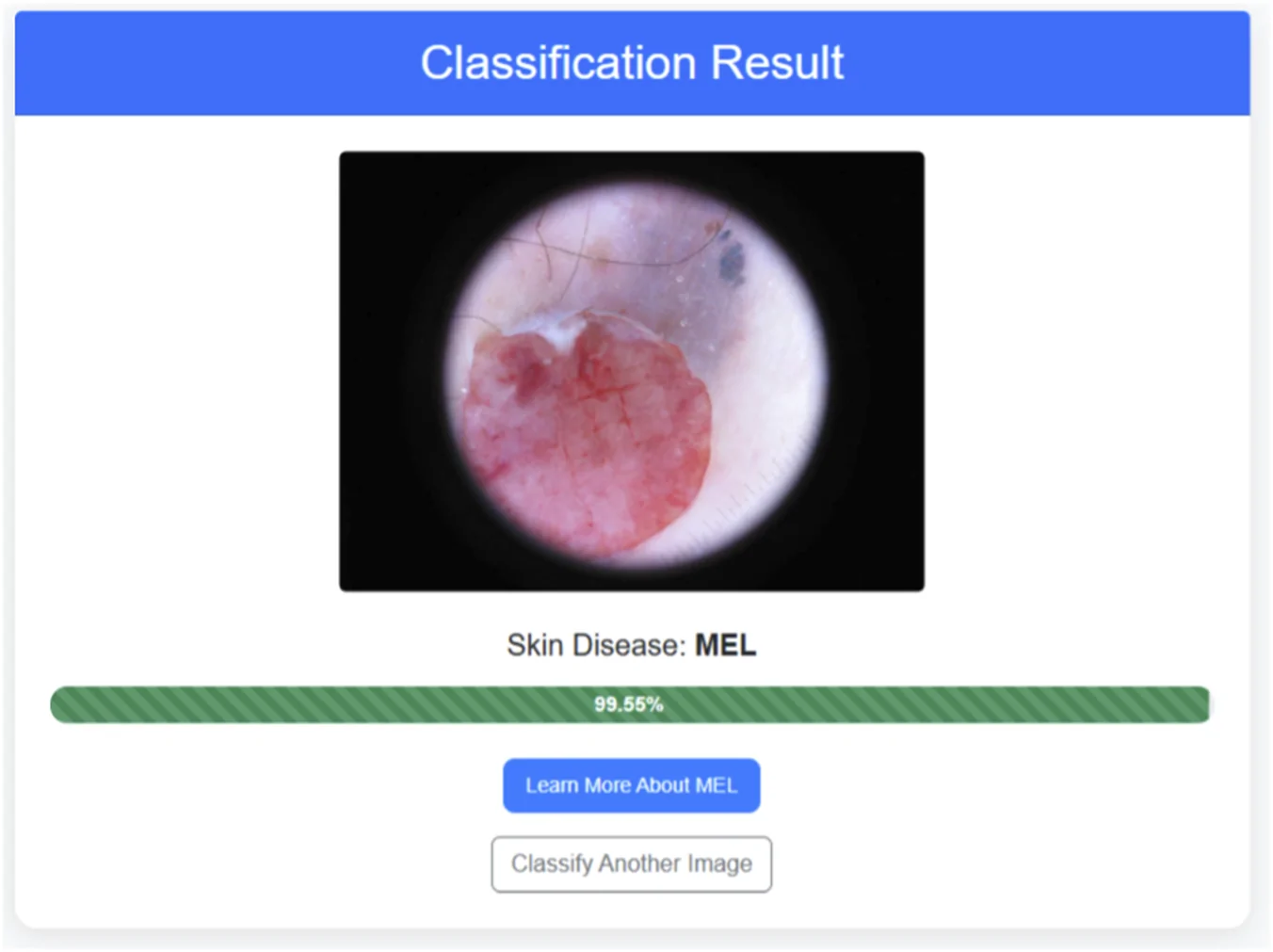
Classification result.

## 7 Theoretical framework

This section discusses four theoretical views: ensemble learning, semantic search, generalizability and clinical decision theory, and explainable AI theory.

### 7.1 Ensemble learning theory

The theoretical insights of ensemble learning demonstrate its success and show that, according to diversity theory, applying different models within the same architecture can achieve better results. The results of the proposed architecture demonstrate this, indicating that the use of CNN and CNN-LSTM together as an ensemble captures both spatial features and sequential dependencies, resulting in more accurate classification than single models.

### 7.2 Semantic search theory

Semantic search reasoning provides better results than rule-based probabilistic theory. It is based on feature embeddings of images rather than predefined rules or probability estimations. Therefore, the results indicate that employing feature embeddings can effectively improve both skin lesion classification and semantic image retrieval by retrieving similar cases, even when their exact appearances differ.

### 7.3 Generalizability and clinical decision theory

Although a single dataset is used in the proposed architecture, it achieved a high-performance classification result. This system provides a decision-support framework for recommendations alongside explanations to support clinicians in making final decisions by integrating explainability and semantic search retrieval.

### 7.4 Explainable AI theory

The proposed architecture integrates local explanations represented by Grad-CAM with example-based explanations represented by semantic search, aligning with the human decision metric to provide more understanding and evidence-based reasoning to help clinical decision theory. Grad-CAM highlights the lesion areas, while semantic search retrieves similar cases based on their feature embeddings.

## 8 Performance comparison

This section presents a comparison of the results obtained from several models: a CNN, a hybrid CNN–LSTM architecture, a stacking ensemble classifier evaluated using accuracy, and AUC metrics (Figure 18). The comparison indicates that the stacking ensemble approach improves the performance of deep learning (DL) models. As shown in the figure, the CNN model achieved its highest performance with an accuracy of 0.97 and an AUC of 0.9987, while the hybrid CNN–LSTM model reached an accuracy of 0.95 and an AUC-ROC of 0.9888. The stacking ensemble model using XGBoost outperformed both models, achieving an accuracy of 0.9733 and an AUC of 0.9968. A further comparison with recent studies employing the same ISIC 2019 dataset, along with other datasets evaluated using similar performance metrics, is presented in Tables 6 and 7 and Figure 19. The results indicate that these models achieve competitive performance compared with existing studies. Some existing studies report a higher accuracy on ISIC 2019. These differences are likely due to possibly be different train/test splits, dataset preprocessing, and the use of very large models, which may lead to overfitting. Most of the exited approaches in the literature rely on pre-trained deep learning architecture.

**FIGURE 18:**
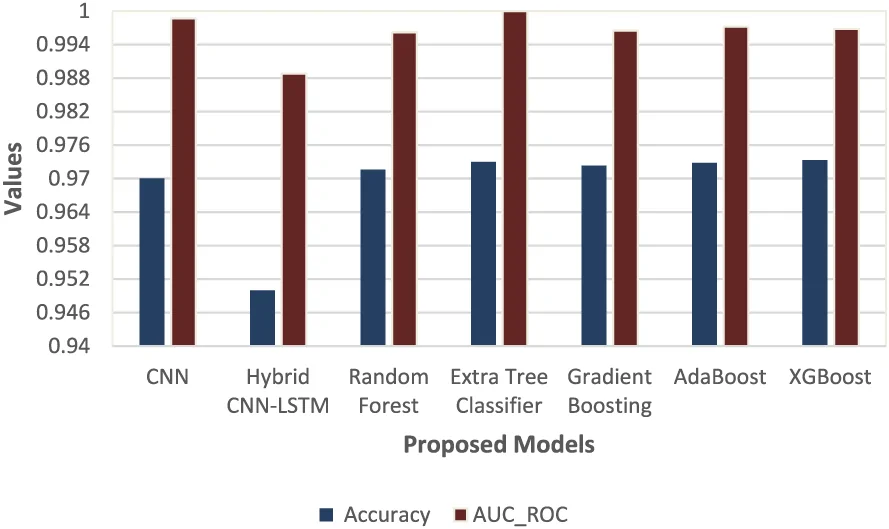
Comparison between results of proposed models.

**TABLE 6:** comparison of proposed model with other related research on ISIC 2019 classification.

Source	Model	Dataset	Accuracy
(Bipin et al., 2026)	Hybrid (ResNet + ConvNeXtV2 + ViT-B/16)	ISIC 2019	95.6%
(Ozdemir and Pacal, 2025)	ConvNeXtV2+	ISIC 2019	93.48%
(Aboulmira et al., 2025)	Dempster–Shafer fusion and EfficientNet	ISIC 2019	84.67%
(Saha et al., 2024)	SwinViT	ISIC 2019	99.97%
(Houssein et al., 2024)	DCNN	ISIC 2019	91.1%
(Ahammed et al., 2022)	SVM	ISIC 2019	95%
(Khan et al., 2025)	CAD-Skin (CNN and autoencoders + QSVM)	ISIC 2019	99%
(Aruk et al., 2025)	Hybrid ConvNeXt (CNN + ViT)	ISIC 2019	92.50%
(Pranav and Sarath, 2023)	ConvNeXt	ISIC 2019	87.37%
(Nugroho et al., 2023)	Inception-V3	ISIC 2019	96.40%
Proposed CNN Model	CNN	ISIC 2019	97%
Proposed Hybrid CNN-LSTM Model	Hybrid CNN with LSTM		95%
Proposed Ensemble Stacking Model	Meta-Model: XGBoost		97.33

**TABLE 7:** Comparison of proposed model with other related research on other datasets on skin disease classification.

Source	Model	Dataset	Accuracy
(Jimi et al., 2025)	Hybrid Swin Transformer + CNN	ISIC 2016	98.25%
		ISIC 2017	98.30%
		ISIC 2018	97.89%
(Najjar et al., 2025)	VGG19-RSPDA-ViT (VGG19+ViT)	MSK10000	97.9%
		HAM10000	97.1%
		PH2	98.67%
(Aboulmira et al., 2025)	Dempster–Shafer fusion and EfficientNet	ISIC 2020	98.57%
		HAM10000	94.8%
		PAD-UFES-20	90.87%
(Padhy et al., 2025)	Hybrid R-LSTM50 (ResNet-50_LSTM)	ISIC 2020	95.72%
		HAM10000	94.23
(Ahmad et al., 2025)	CNN	ISIC 2020	94.5%
(Vivek and Ramesh, 2025)	ResNet50, InceptionV3, and VGG16	HAM10000	87.5%
(Khan et al., 2025)	CAD-Skin (CNN and autoencoders + QSVM)	PAD-UFES-20-Modified	98%
		ISIC-2018	99%
(Sarı and Keser, 2025)	AlexNet relief	Kaggle dataset	92.10%
		ISIC 2017	91.11%
(Aruk et al., 2025)	Hybrid ConvNeXt (CNN + ViT)	HAM10000	94.30%
Proposed CNN Model	CNN	ISIC 2019	97%
Proposed Hybrid CNN-LSTM Model	Hybrid CNN with LSTM		95%
Proposed Ensemble Stacking Model	Meta-Model: XGBoost		97.33

**FIGURE 19:**
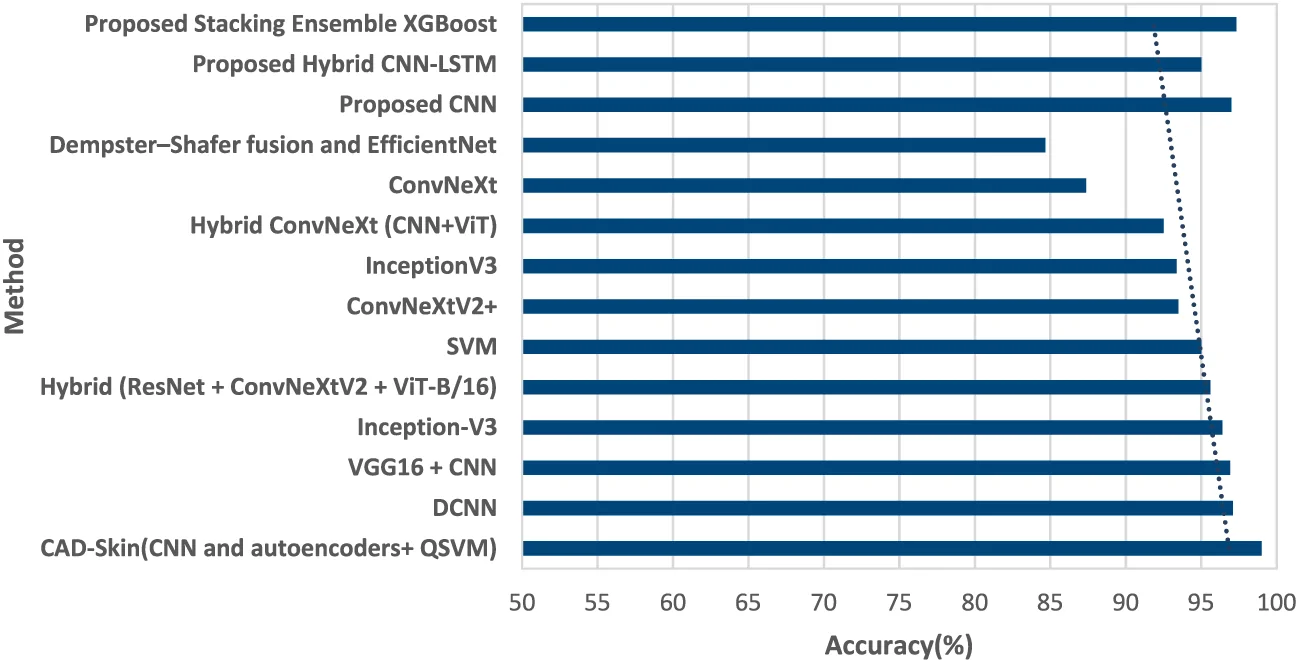
Comparison graph of the proposed model with other existing studies on the ISIC 2019 dataset.

## 9 Discussion

This study evaluates the performance of three models on the ISIC 2019 dataset, in which the stacking ensemble model achieved the best results. This improvement can be attributed to several factors. First, different models are likely to learn different features of the data, so combining their predictions creates an ensemble effect that improves both generalization and robustness. This is mainly useful in skin lesion detection, where the visual differences between lesions are small, making them difficult to detect, and ensemble models often perform better than individual models. Second, integrating multiple DL models produces an accurate prediction compared to single models. Additionally, data augmentation was applied to address the dataset’s class imbalance; it did not affect the results of the models. The high AUC-ROC values, confusion matrices, and macro-averaged metrics show that the models achieved the best results using this data augmentation, which improved the representation of minority classes. In addition, semantic search-based feature embedding was used to retrieve images that share the same features, which supports the interpretation and validation of the model’s predictions. To enhance the model’s transparency, explainability methods such as Grad-CAM and Grad-CAM++ were applied. The results show strong and competitive performance compared to several existing studies on the ISIC 2019 dataset.

## 10 Study limitations

This study has several limitations.

- Data limitations include dataset bias, class imbalance, and generalizability concerns, because the framework was evaluated using a single dataset. Although data augmentation was applied to address the class imbalance, the resulting balanced data is based on the augmented images, and it may not perfectly represent the real-world clinical data.
- Methodological limitations relate to the selection and design of the proposed architecture according to the characteristics and class distribution of the current dataset. The preprocessing techniques were also designed according to the characteristics of the ISIC 2019 dataset. Therefore, evaluating the same architecture on another dataset may require modifications to the architecture and preprocessing techniques as well as retraining the model to accommodate differences in the characteristics, class distribution, and clinical categories of other datasets.
- Clinical limitations concern the need for practical deployment to ensure that all challenges and validation gaps that may occur after clinical validation are addressed. The system has not yet undergone any kind of external validation, so it is not ready to be a diagnostic tool. It needs further validation before deployment in actual clinical practice.
- Technical limitations include the computational and implementation requirements of the proposed architecture, which integrate multiple components such as DL models, AI explainability, and semantic search. Although the CNN is lightweight and supports the real time inference of CPU, integrating it with CNN–LSTM, stacking ensemble, explainable AI techniques, and semantic search may need additional computational and implementation requirements to be considered during deployment.

## 11 Conclusion and future research

The proposed architecture achieved a high performance on the ISIC 2019 dataset by employing the CNN, hybrid CNN–LSTM, ensemble stacking classifiers, and a semantic search-based feature embedding. The ensemble stacking with XGBoost as a meta model achieved the highest accuracy score of 0.9733 and an AUC score of 0.9968. The semantic search approach and the Grad-CAM enhanced the model’s interpretability and reliability. Based on the results of the proposed models, a web-based classification system for skin lesion classification is introduced to help patients and clinicians in diagnosis. Beyond its experimental performance, the proposed model has several real-world applications. The web-based system can be used as a clinical decision-support tool. It can provide preliminary classification for skin lesion images, similar case-based lesion through semantic search, and visual explanations using Grad-CAM and Grad-CAM++ techniques. It can also support patients and clinicians in remote screening by enabling rapid classification for skin lesion images through a web interface. Moreover, its lightweight architecture and real time inference on CPU make it suitable for any healthcare where GPU resources are not available and hardware resources are limited. Importantly, these applications are designed as potential decision-support tools rather than for self-diagnosis, as the system has not yet undergone external or clinical validation. Future research will focus on addressing the study’s limitations. First, the proposed architecture will be evaluated on external datasets, such as HAM10000 and PH2, to evaluate its generalizability across different dataset characteristics. Such evaluations may require appropriate architectural modifications and model retraining. Second, practical deployment will be investigated to assess system applicability and address potential validation gaps. Third, computational and implementation strategies will be explored to improve the model’s efficiency. Finally, multimodal clinical information and privacy-preserving approaches, including federated learning, will be investigated to support more robust and secure multi-center model development.

## Data Availability

The dataset(s) used during the current study are publicly available at: https://www.kaggle.com/datasets/salviohexia/isic-2019-skin-lesion-images-for-classification.
